# Nano-Silica as Designer Tools for Geopolymer Microstructure Optimization: Effects on Porosity, Interfacial Transition Zone (ITZ), and Mechanical Performance

**DOI:** 10.3390/ma19112320

**Published:** 2026-05-31

**Authors:** Kinga Korniejenko, Qinglin Wu

**Affiliations:** 1 Faculty of Material Engineering and Physics, Cracow University of Technology, Jana Pawła II 37, 31-864 Cracow, Poland; 2 School of Renewable Natural Resources, Louisiana State University AgCenter, Baton Rouge, LA 70803, USA; qwu@agcenter.lsu.edu

**Keywords:** geopolymer, nano-silica, microstructure

## Abstract

**Highlights:**

- Nano-silica (NS) acts as an active design tool controlling geopolymer microstructure, porosity, and interfacial transition zone (ITZ) performance.
- Properly dispersed NS enhances strength, durability, and early geopolymerization kinetics at low dosages.
- NS refines pore structure and transforms the ITZ into a dense, load-bearing zone in geopolymer composites.
- The effects of NS are highly system- and dosage-dependent, with no universal optimal addition level.
- NS enables advanced geopolymer applications, including 3D printing, ITZ engineering, and In Situ Resource Utilization (ISRU) materials.

**Abstract:**

Nano-silica (nano-SiO_2_) has emerged as a powerful designer tool for engineering the microstructure of geopolymer composites, enabling precise control over porosity, interfacial transition zone (ITZ) characteristics, and resultant mechanical performance. The main aim of this review is to evaluate the role of nano-silica as a reinforcement and pozzolanic accelerator. The paper delivers a critical literature overview. It is based on a comprehensive critical review of the existing literature and illustrative case studies demonstrating practical applications in geopolymer composites. The article presents the key mechanisms connected with the application of nano-additives, including accelerated geopolymerization kinetics and heterogeneous nucleation on nano-silica surfaces. Comprehensive characterization methods are critically assessed, including SEM/EDS for gel morphology, MIP for porosity profiles, XRD/FTIR for reaction products, micro-CT for 3D void networks, and nanoindentation for ITZ mechanical gradients. The article also shows the main applications span high-performance concretes, 3D-printed geopolymer elements (improved buildability and interlayer adhesion), and durable overlays. The article is a closed presentation of challenges such as long-term stability, alongside future directions. The main findings show that nano-silica offers a pathway to tailored, low-carbon geopolymers with superior microstructure–performance relationships aligned with sustainable construction goals.

## 1. Introduction

The incorporation of nanomaterials significantly enhances the mechanical and durability properties of geopolymer concrete, often allowing the reduction or elimination of energy-intensive heat curing. Nanomaterials promote matrix densification by refining the microstructure, reducing porosity and water absorption, and improving resistance to aggressive environments such as acids, sulfates, and chlorides [1]. They can significantly redefine the properties of the geopolymer materials and give them new perspectives for applications [2].

Among the various nanomaterials incorporated into geopolymer concrete, nano-silica (NS) is the most widely reported and systematically studied, with consistent evidence of its influence on gel densification, pore refinement, and residual mechanical performance after thermal exposure [3]. NS has been identified as the most efficient and versatile nano-additive for geopolymer concrete [1,4]. It is also the most widely recognized nano-additive in geopolymers [5,6]. It is because NS stands out as offering the best balance between performance, availability, and cost, while also exhibiting a broad range of experimentally validated effects [7,8].

NS is one of the most effective nano-additives for geopolymers, provided it is dosed precisely. Its action is dual in nature: physical (pore filling) and chemical (intensification of geopolymerization) [9]. The addition of NS leads to densification and homogenization of the geopolymer concrete microstructure through a nano-filler effect, acceleration of geopolymerization reactions, and increased formation and continuity of N-A-S-H/C-A-S-H gels, resulting in reduced pores and microcracks and a strengthened interfacial transition zone (ITZ) [4,10]. Compared to inert nanoparticles, NS offers significantly greater potential for enhancing the load-bearing capacity and durability of geopolymers, although it requires control over agglomeration and the increase in brittleness observed at higher dosages [9,11].

The research on the influence of NS for geopolymers has a particular meaning, because it shows a stronger effect in geopolymer concretes than in ordinary Portland cement (OPC) systems due to the highly alkaline environment [12]. NS acts as a designer tool rather than a passive filler, since it actively modifies the geopolymerization process by increasing soluble silica availability, accelerating reaction kinetics, and refining the gel network [13,14].

Despite the growing number of studies on NS in geopolymer systems, the current literature remains fragmented, with most works focusing on isolated properties such as mechanical strength or durability, often without a unified interpretation of the underlying mechanisms. In particular, there is a lack of comprehensive analyses linking nanoscale processes—such as nucleation, gel formation, and silica reactivity—with mesoscale features including pore structure refinement and interfacial transition zone (ITZ) behavior, and ultimately with macroscopic performance.

Therefore, the main objective of this review is to address this gap by presenting NS as an active microstructure design tool rather than a passive additive. The novelty of this work lies in the integration of multi-scale mechanisms governing NS action, with particular emphasis on the role of NS in controlling porosity, tailoring ITZ properties, and optimizing mechanical performance. In addition, this review combines a critical evaluation of the recent literature with selected illustrative case studies to demonstrate how these mechanisms translate into practical applications, including advanced composites and emerging technologies such as 3D printing. By structuring the discussion around mechanism–structure–performance relationships, this article provides a more coherent framework for understanding and designing NS-modified geopolymer systems, thereby contributing to the development of high-performance and sustainable construction materials. The article also shows the current limitations and future directions. The main findings indicate that NS enables the development of tailored geopolymers with enhanced microstructure–performance relationships, supporting advanced and sustainable construction applications.

## 2. Literature Review Methodology

**Figure 1:**
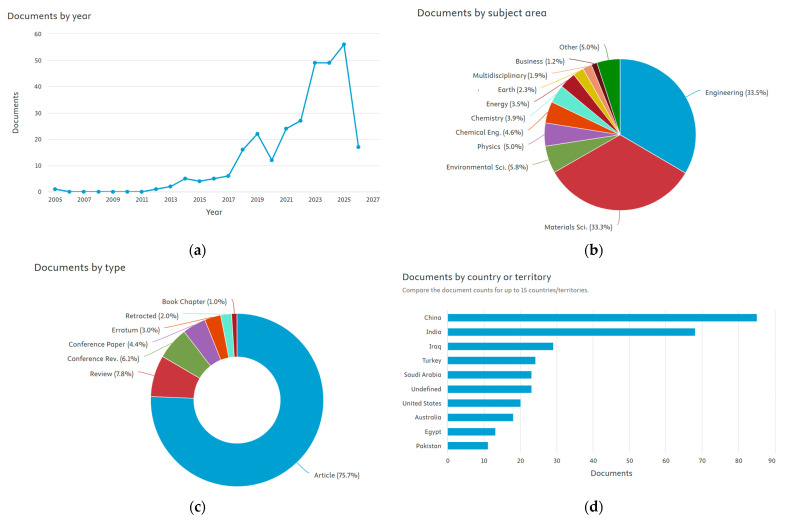
The findings of the literature search conducted in the Scopus database: (**a**) number of documents by year; (**b**) analysis of documents by subject; (**c**) analysis of documents by type; (**d**) number of documents by country [15].

The methodology adopted in this article is founded on a critical review of the scientific literature and selected relevant case studies. In the initial phase, two keywords were systematically combined to retrieve pertinent records from the Scopus database. The following keywords were used: “geopolymers” and “nano silica”. Figure 1 shows the results for this query.

The Scopus database pointed to 296 relevant results. It should be noted that the number of retrieved publications is influenced by the specific keyword combination (“geopolymers” AND “nano silica”), which was intentionally selected to ensure a focused and highly relevant dataset. Broader search terms, including other nanomaterials, would yield a larger number of results; however, such an approach would reduce thematic consistency and obscure the specific role of nano-silica. Therefore, the presented analysis reflects a targeted subset of the literature dedicated specifically to NS-modified geopolymer systems rather than the broader field of nano-modified materials.

The analysis of the results shows that the topic is very new, with the first publications registered in the database from 2005 (Figure 1a). It is also worth noting that the earliest studies on the incorporation of nano-silica into geopolymer systems appeared shortly after the broader introduction of nano-modification concepts in cementitious materials. However, it is worth noting that after this single publication there was quite a long break and the next publication appeared in 2012. These initial works focused primarily on identifying basic effects such as microstructural densification and mechanical enhancement, and laid the foundation for subsequent research development. So, in fact, this research topic has been developed for less than 15 years (Figure 1a). The relatively slow growth observed until around 2016 can be attributed to the emerging nature of both geopolymer and nanomaterial research at that time, as well as the limited availability and higher cost of nano-silica. The noticeable increase in publications after 2017 reflects a convergence of several factors, including the rapid advancement of nanotechnology, improved commercial accessibility of nano-silica, and the growing global emphasis on sustainable and low-carbon construction materials. In particular, the increasing adoption of geopolymers as alternatives to Portland cement and the development of advanced characterization techniques have significantly contributed to the expansion of research activity in this area. Interest in this topic has shown a sustained upward trend, aside from a temporary disruption during the COVID-19 pandemic in 2020. In the last few years, about 50 articles have been published in this area annually (Figure 1a).

The subject-area analysis reveals the interdisciplinary nature of this topic, with engineering and materials science representing the two most prominent domains (Figure 1b). Most of the publications are research articles, more than 75%. The share of review papers is 7.8%. That shows a potential for development of the review studies in this area (Figure 1b). The largest number of publications is from India and China, which is quite typical in current science (Figure 1d).

**Figure 2:**
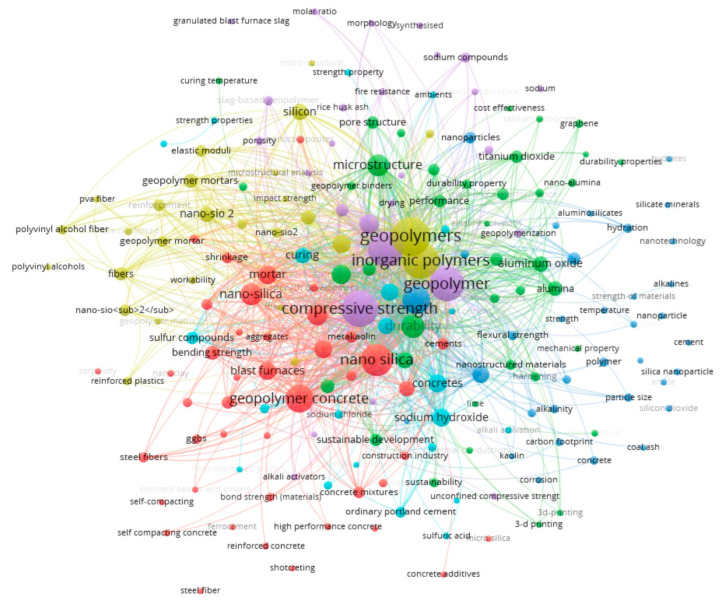
A visualization of the network of connections based on keywords used in the articles, generated using VOSviewer.

To enhance the visualization of the research domains relevant to the scope of this article, VOSviewer software (version 1.6.20; Centre for Science and Technology Studies, Leiden University, Leiden, The Netherlands) was employed. The analysis was based on an Excel datasheet containing 296 records retrieved from the Scopus database. The resulting map is presented in Figure 2.

The visible keywords and identified connections show a wide range of provided investigations. They also show that among the properties of geopolymers, the main attention is focused on compressive strength, which is the main reason for modifying the geopolymer by NS.

From the retrieved publications, the authors selected those most relevant in terms of content for the respective sections of this article. The literature review placed particular emphasis on publications from the last five years, covering the period from January 2020 to March/April 2026.

## 3. Geopolymer Microstructure in the Context of Nano-Silica Modification

Geopolymerization proceeds through successive dissolution–transport–polycondensation stages, beginning with the alkaline dissolution of aluminosilicate precursors and the release of reactive Si and Al monomers. These species are subsequently transported in the pore solution and undergo polycondensation to form a three-dimensional inorganic polymer network dominated by Si–O–Al (S–O–T) linkages [16]. In low-calcium systems, this process primarily yields an amorphous N-A-S-H gel, characterized by a highly cross-linked aluminosilicate framework that governs mechanical strength. In calcium-containing systems, partial substitution leads to the formation of C-(N)-A-S-H gel, where Ca participates in the gel structure, resulting in a hybrid network with modified chain connectivity, higher density, and distinct structural and mechanical characteristics [16]. To better contextualize the role of nano-silica (NS), it is important to distinguish the microstructure of geopolymers produced with and without its addition. In unmodified systems, the microstructure is typically characterized by a relatively heterogeneous gel distribution, the presence of larger capillary pores, and a less uniform interfacial transition zone (ITZ). In contrast, NS-modified geopolymers exhibit a more homogeneous and compact microstructure, with a refined pore structure, reduced pore connectivity, and improved gel continuity. This difference arises from the dual action of NS, which acts both as a reactive silica source and as a nano-filler, promoting densification and enhancing the formation of N-A-S-H and C-(N)-A-S-H gels.

Recent studies on NS incorporation further refine the geopolymerization process by modifying the dissolution–polycondensation balance and the resulting gel chemistry [16]. NS acts as a highly reactive silicon source that enhances early Si availability, promotes Si–O–Al bond formation, and accelerates the development of N-A-S-H gel when the Si/Al ratio is properly balanced. However, excessive NS can disrupt Al dissolution and hinder polycondensation, leading to silica-rich by-products rather than a continuous N-A-S-H network. In calcium-bearing systems, NS additionally influences the coexistence and structural integration of C-(N)-A-S-H gel, affecting gel density, chain connectivity, and long-term mechanical performance [16]. Javed et al. [17] provided a quantitative distinction between geopolymer gels, identifying C-(N)-A-S-H as the primary load-bearing phase, while N-(C)-A-S-H is shown to be a secondary and structurally weaker gel. Consequently, it demonstrates that effective geopolymer design with NS requires controlling the type of gel formed rather than simply maximizing the SiO_2_ content. The influence of NS on the geopolymer microstructure is also strongly dependent on its dosage. At low and optimal contents, NS is well dispersed within the matrix and effectively promotes nucleation, gel formation, and pore refinement, leading to a denser and more uniform structure. However, excessive NS content may result in particle agglomeration, which limits its reactivity and creates localized defects and secondary porosity. Consequently, rather than continuously improving the microstructure, high NS dosages may disrupt the homogeneity of the gel network and reduce the efficiency of geopolymerization. Therefore, the relationship between NS content and the microstructure should be considered non-linear and system-dependent. The mechanism of formation was also confirmed by atomic-scale radial distribution function analysis, which revealed distinct Si–O bonds between NS and aluminosilicate oligomers, as well as Al–O and Na–O interactions involving oxygen atoms originating from the silica phase [18]. The matching bond lengths and coordination environments demonstrated that Si and O atoms from NS are directly incorporated into the N-A-S-H framework, yielding interfacial reaction products with the same chemical nature as conventional N-A-S-H gel [18].

In practice, these changes in the geopolymer microstructure can also be confirmed by backscattered electron imaging. The observation made by Luo et al. [19] shows that this process is also dynamic in time and after 28 days, the proportion of N-A-S-H gel is comparable in both reference and nano-modified geopolymers, typically ranging from about 49% to 56%, indicating that the addition of NS does not lead to a substantial increase in the total gel content at a mature age. They therefore emphasize that the observed improvements in macroscopic strength are not governed by the quantity of gel formed, but rather by changes in its quality and microstructural arrangement [19].

A slightly different mechanism connected with NS has been observed in phosphoric acid-activated geopolymers [20,21]. In this kind of geopolymer, NS acts as a highly reactive silicon source in the acidic (phosphoric acid) environment, where it readily dissolves and supplies silanol species that actively participate in geopolymerization [22]. It promotes the condensation of Si–OH groups into stronger Si–O–Si and Si–O–P linkages, thereby reinforcing the Al–O–P and Si–O–P backbone of the phosphate geopolymer network [22].

The confirmation of this is changes in the micromechanical properties of N-A-S-H gel. Nanoindentation results demonstrate that NS enhances the micromechanical properties of the N-A-S-H gel, increasing both its elastic modulus and hardness compared with the reference geopolymer. In particular, the elastic modulus of the gel increases from approximately 11.0 GPa in the reference sample to about 13.3 GPa in the NS-modified system, reflecting a mechanically stiffer and more robust gel phase [19].

In addition to composition and NS content, synthesis and curing parameters play a critical role in determining the final microstructure of NS-modified geopolymers. Factors such as curing temperature, pressure conditions, and the presence or absence of saturated environments significantly influence dissolution kinetics, gel formation, and pore development. Elevated curing temperatures generally accelerate geopolymerization and enhance early microstructural densification, while ambient curing conditions require the presence of highly reactive components such as NS to achieve comparable results. Similarly, moisture conditions affect the transport of reactive species and the continuity of gel formation, with insufficient moisture potentially limiting polymerization and leading to increased porosity. Therefore, the microstructure of NS-modified geopolymers results from a complex interaction between composition, processing conditions, and nanoparticle dispersion.

**Figure 3:**
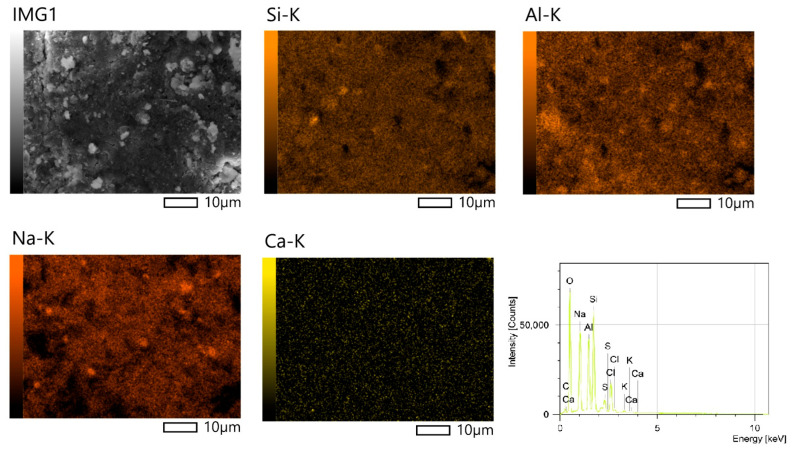
SEM image with elemental maps and EDS spectrum.

The useful information can also be delivered by SEM observation. Figure 3 shows an exemplary spatial distribution of major elements and the microstructural integration of NS investigated by SEM imaging and EDS mapping. Figure 3 was made by the authors using JSM-IT200 InTouchScope™ SEM (JEOL, Tokyo, Japan). The geopolymer samples with 10% by vol. NS were coated with a thin layer of gold and placed in a carbon pot to ensure electrical conductivity. The SEM observation was made on samples after mechanical tests.

Based on SEM-EDS elemental mapping, the spatial distribution of major elements within the NS-modified geopolymer matrix can be observed (Figure 3). SEM image reveals a relatively homogeneous binder phase with no pronounced contrast associated with discrete silica-rich particles, suggesting the absence of unreacted NS agglomerates at the microscale. The elemental maps of silicon and aluminum show a highly overlapping and uniform distribution, indicating the formation of a continuous aluminosilicate gel network. The concurrent presence of sodium within the same regions supports the identification of an N-A-S-H-type gel as the dominant binding phase. Importantly, no isolated silicon-rich domains were detected, implying that the added NS does not persist as a distinct phase but is incorporated into the geopolymer gel structure. The corresponding EDS spectrum acquired from the analyzed area (Figure 3) confirms the dominance of Si, Al, O and Na, with minor Ca contribution, consistent with the elemental maps. Overall, these observations demonstrate that NS acts as an integrated reactive component of the geopolymer matrix, contributing to gel densification and structural uniformity rather than increasing the total gel content through phase separation.

The homogeneous distribution of Si and Al observed in the SEM-EDS maps, together with the absence of isolated silica-rich domains, is consistent with previous studies reporting the structural incorporation of NS into the geopolymer gel rather than the formation of a separate SiO_2_ phase. Similar microstructural features, including gel densification and improved phase integration, have been widely documented for NS-modified geopolymers in the literature [23,24].

Other important microstructural aspects are observations of crystallinity and mineral phases. Paruthi et al. [25] analyzed XRD changes connected with the incorporation of nano-SiO_2_, which leads to noticeable changes in the intensity of silicate-related peaks, and in some geopolymer systems, the formation of an additional calcium silicate carbonate phase is reported [25]. However, NS does not consistently result in the emergence of new crystalline phases; instead, its primary effect is associated with modifications in the degree of crystallinity and with influencing the progression and intensity of the geopolymerization reaction [25]. Ahmed et al. [26], also in an XRD investigation, did not reveal the formation of new, clearly distinct crystalline phases attributable solely to the addition of nano-SiO_2_, but instead indicated increased intensity of gel phases (C–S–H, C–A–S–H, N–A–S–H) and a higher contribution of silica [26].

The other confirmation that NS modifies the chemical structure of the geopolymer network by actively participating in geopolymerization rather than acting only as an inert filler can be found in Nuclear Magnetic Resonance (NMR) research [27]. ^29^Si MAS NMR shows a shift and increased intensity of Q^4^ (mAl) species, indicating a higher degree of silicate polymerization and a more cross-linked aluminosilicate network, while ^1^H MAS NMR reflects changes in bonded hydroxyl groups consistent with stronger Si–O–Al/Si–O–Si connectivity [27]. These NMR results demonstrate that properly dosed NS promotes network densification at the atomic scale [27].

## 4. Nano-Silica: Properties and Design Parameters

### 4.1. Types of Nano-Silica Used in Geopolymers

NS can be defined as amorphous silicon dioxide (SiO_2_) particles with characteristic dimensions in the nanometer scale, typically below 100 nm, exhibiting a high specific surface area and enhanced chemical reactivity compared to bulk silica [28,29]. NS differs from micro-silica primarily in its much smaller particle size and higher surface reactivity, which allow it to actively participate in geopolymerization and modify the gel network at the molecular level. In contrast, micro-silica acts mainly as a filler and a source of crystalline reinforcement, promoting densification and hardness through physical packing and phase formation rather than extensive chemical interaction [22]. The most commonly applied types of NS used as an additive for the geopolymer matrix are presented in Table 1.

Except for forms of the NS mentioned in Table 1, some authors also include silica fume (SF) as a type of NS. However, NS differs from SF primarily in particle size and surface area. Silica fume consists of amorphous spherical SiO_2_ particles with primary diameters typically ranging from 0.1 to 0.3 µm; however, in practical applications, it is present mainly as micron-scale agglomerates, whereas NS is composed of discrete particles in the 5–100 nm range with substantially higher surface area and reactivity [42,43].

It is worth mentioning that the agglomerated particles of silica fume can be dispersed to a certain extent through tailored processing methods, including high-shear mixing or sonication, although residual micron-scale clusters typically remain [44]. Another method is the calcination of SF at 800 °C, which was shown to enhance the reactivity of SF and to intensify its beneficial effects on geopolymer properties, leading to a denser microstructure and significantly improved mechanical strength and durability of the geopolymer matrix [45].

An interesting observation was made by Paruthi et al. [46]. The combined use of NS and SF proved significantly more effective than the individual incorporation of either additive alone [46]. This synergistic behavior is attributed to the simultaneous chemical contribution of highly reactive silica, which enhances geopolymer gel formation, and the physical pore-filling effect, which leads to a denser and less permeable microstructure. As a result, the NS–SF system provides superior durability and resistance to chemical attack compared with single-component modifications [46].

Colloidal NS and NS in dry powder form are the most widely applied in geopolymers. Colloidal NS, supplied as a stable aqueous dispersion, provides superior particle distribution within the matrix and a pronounced nucleation effect, leading to accelerated early-age reactions; however, its application is limited by higher cost and potential long-term stability issues of the sol. In contrast, dry-powder NS consists of amorphous SiO_2_ nanoparticles with extremely high specific surface area and storage stability, but its effectiveness strongly depends on intensive dispersion techniques due to a high tendency for agglomeration and dosing difficulties [31,47]. More details about these two types are also presented in Section 4.2.

Surface modification of NS is relatively rarely made, because of the increasing cost. However, the application of this method is very widespread and also allows for combining NS with other additives as well as creating advanced composites. Such works were provided by Zhang et al. [48]. They prepared a core–shell structure by acid-leaching copper slag to obtain a rigid Fe-rich core, coating its surface with a PVP interlayer, and subsequently depositing a uniform NS shell via controlled adsorption, forming CS@PVP@NS particles [48]. This composite combines the mechanical load-bearing role of the copper slag core with the high reactivity of the NS shell, enabling effective nucleation of C-(A)-S-H/N-A-S-H gels, pore refinement, and significant improvements in strength and elastic modulus. The prepared powder was added directly to the geopolymer dry mix as a solid additive at a dosage of 2 wt.% of the total solid precursors [48]. By combining a rigid load-bearing core with a reactive NS shell, the system simultaneously improves stress transfer and gel polymerization efficiency, achieving synergistic enhancements that cannot be attained by dispersed NS acting only at the nanoscale. Additionally, this composition prevents NS agglomeration and ensures its uniform chemical anchoring at mechanically active interfaces. Another composition with functionalized NS was developed by Gu et al. [49] Nano-SiO_2_@Fe_3_O_4_ magnetofluid effectively modifies geopolymer matrices by simultaneously enhancing magnetic performance and mechanical strength. In this system, NS acts as a functional carrier that improves the dispersion of Fe_3_O_4_ nanoparticles and provides active nucleation sites for geopolymerization [49]. As a result, NS plays a key multifunctional role by promoting geopolymer gel formation and indirectly stabilizing the electromagnetic behavior of the hybrid geopolymer composite [49].

A modern approach for obtaining in situ NS was proposed by Sun et al. [38]. The carbonation-mediated in situ growth method is based on CO_2_-induced precipitation of NS from a sodium silicate solution, where activated fly ash particles serve as substrates for preferential heterogeneous nucleation [38]. The process involves fly ash surface pretreatment, controlled carbonation to trigger NS growth directly on particle surfaces, and simple post-processing. This approach yields uniformly dispersed, chemically anchored NS that avoids agglomeration, enhances geopolymerization, improves strength and durability under ambient curing, and offers a cost-effective, energy-efficient, and scalable alternative to conventional NS addition methods [38]. Moreover, compared to commercial NS, which consists of freely dispersed particles with a similar nominal size (~50 ± 5 nm) but a strong tendency to agglomerate in the highly alkaline geopolymer environment, in situ-grown NS is chemically anchored to fly ash surfaces through Si–O–Si bonds, ensuring stable dispersion. As a result, in situ-grown NS provides higher compressive strength enhancement, reduced workability loss, lower variability, and fewer microstructural defects than commercial NS, particularly at higher dosages [38].

Currently, one of the most actively investigated research directions is the development of waste-derived and bio-derived NS, driven by the need to reduce the environmental footprint and energy intensity of conventional NS production while simultaneously promoting the valorization of industrial by-products and renewable biomass within a circular-economy framework. Ahmed et al. [50] showed that NS can be synthesized locally from olivine rock using a low-temperature hydrothermal/acid dissolution process (~90 °C), which significantly reduces energy demand compared to conventional high-temperature routes such as flame hydrolysis. The synthesized NS exhibited particle sizes of approximately 7.9–43.4 nm and an amorphous structure, while relying on a naturally abundant raw material and avoiding energy-intensive processing, indicating clear environmental advantages [50]. Importantly, the study directly compares the locally manufactured NS with commercial NS, showing that both provide comparable improvements in the compressive strength of geopolymer concrete, although with slightly different optimal dosages [50]. These results confirm that NS does not need to be expensive or imported to be effective, and that olivine-based green synthesis enables a locally sourced, more sustainable nanomodifier for construction applications. A similar idea was behind the work of Jaddan and Jaber [51]. They replaced NS with nano-metakaolin (~93 nm). It acts as an ultrafine, highly reactive source of silica and alumina, effectively mimicking the role of NS due to its large specific surface area and rapid dissolution in alkaline media [51].

NS can also be derived from waste streams—such as packaging glass, glass bottle waste, or silica-rich ashes—and has emerged as a sustainable alternative to commercial NS. When used in geopolymer systems, waste-derived NS can provide comparable nucleation and pore-refining effects while reducing cost and environmental footprint [52]. Their et al. [40] confirm that nano-recycled glass, acting as an NS–like additive, does not alter the binder-controlled hierarchy of geopolymer concrete performance, yet consistently refines the microstructure, enhances gel continuity, and improves resistance to chemical attack by reducing pore connectivity and transport pathways [40]. Its primary contribution lies at the nano- and microscales, where matrix densification and nucleation effects translate into modest but reliable improvements in strength retention under aggressive environments [40].

Bio-based NS is commonly produced from silica-rich agricultural residues, such as rice husk ash, and typically exhibits an amorphous structure with variable particle size and purity depending on the synthesis route and precursor quality. The main advantages of bio-derived NS include its low production cost and reduced environmental footprint, as it enables the valorization of agricultural waste and supports circular-economy strategies. However, the inherent variability of feedstock composition often leads to limited reproducibility and greater scatter in material properties compared to conventional NS [39,53].

It should be noted that the scalability and practical implementation of these different types of NS, including their potential for industrial or pilot-scale applications, are further discussed in later sections of this review, particularly in the context of application-oriented case studies and practical challenges.

### 4.2. Dosage Strategies and Incorporation Methods

In the review article, Vignesh et al. [54] indicate that the optimal NS dosage (typically 1–6 wt.% depending on the system) significantly enhances compressive, flexural, and tensile strength, improves resistance to chemical attack (chlorides, sulfates, and acids), and increases durability under freeze–thaw cycles and cyclic loading [54]. However, excessive NS addition leads to particle agglomeration, reduced workability, and the formation of microstructural defects, highlighting the need for precise control of matrix composition and nanoparticle dispersion [54].

In turn, the study provided by Wang et al. [55] shows that NS exhibits an optimum dosage at a significantly lower value—approximately 0.3 wt.%, at which both the mechanical strength and erosion resistance of geopolymer grouting materials are maximized; further increases in NS content reduce workability and promote particle agglomeration, thereby limiting dispersion efficiency and diminishing the beneficial microstructural effects [55]. Yang et al. [56] confirm that effectiveness is strongly dependent on the synthesis route, dispersion quality, and dosage. The optimal performance was achieved with alkali-catalyzed NS at a low content of approximately 0.16 wt% SiO_2_, which ensured uniform dispersion, dense gel formation, and maximum strength enhancement. In contrast, a higher dosage (0.32 wt%) promoted particle agglomeration, leading to reduced microstructural homogeneity, limited strength development, and the formation of localized defects such as secondary porosity [56]. Similarly, Wang et al. [57] identified an optimal NS addition of about 0.75 wt.% as the most effective level, at which the geopolymer exhibits maximum strength enhancement and microstructural densification. Lower dosages provide limited improvement, while higher dosages lead to nanoparticle agglomeration, increased defects, and a deterioration in mechanical performance [57].

Other values for the dosage of NS are reported by Alagarsamy et al. [58]. They find that at low and optimal dosages, NS is well dispersed in the geopolymer matrix, reacts with aluminosilicate species, accelerates the formation of C-(A)-S-H gels, and efficiently fills capillary and nanoscale pores, leading to a denser and better-connected microstructure. When the NS content exceeds a critical threshold (≈3% by weight of binder), particle agglomeration occurs, resulting in unreacted SiO_2_ clusters and the formation of localized weak zones and voids [58]. Consequently, the matrix polymerization and gel interconnection are effectively reduced, which limits further improvements in mechanical and durability performance [58]. In turn, after analysis of many articles, Indwar et al. [1] claim that an optimal dosage of approximately 2 wt.% of the binder provides the highest compressive strength (up to ~70–75 MPa), the best durability performance, and a favorable balance between technical benefits and material cost [1].

The other approach is represented by Jaddan and Jaber [51]. They claim that its performance is governed by an optimal Si/Al balance rather than silica content alone, as excess silica remains unreacted as SiO_2_ (quartz) and weakens the geopolymer structure [51]. This approach is also supported by the investigation provided by Choi et al. [59]. They point to an optimal silicon-to-aluminum ratio of approximately 1.8, at which the geopolymer exhibited the highest compressive strength and the most homogeneous N-A-S-H gel structure [59].

In turn, Shumuye et al. [60] pointed out that the amount of NS is not the only factor that influences the properties, but also the size of NS particles is crucial [60]. NS significantly enhances the mechanical performance of engineered geopolymer composites only when both dosage and particle size are optimized, with the best results achieved at 1% NS and an average particle size of ~15 nm, where the compressive strength reached 94.2 MPa after 28 days, outperforming all other compositions [60]. The particle size of NS is as critical as its content, since 15 nm particles provide superior pore filling and higher reactivity, leading to effective microstructural densification. In contrast, larger NS particles (50–100 nm) exhibit reduced reactivity and a weaker densification effect, resulting in lower compressive strength due to less efficient pore refinement [60].

It should be emphasized that the wide range of reported optimal NS dosages in the literature does not indicate inconsistency, but rather reflects the strongly system-dependent nature of geopolymer materials. The effectiveness of NS is governed by multiple interacting parameters, including precursor chemistry (e.g., fly ash, GGBFS, metakaolin), calcium content, Si/Al ratio, alkalinity, and—critically—the dispersion quality of the nanoparticles. As a result, NS addition exhibits a non-linear behavior: at low and optimal contents, it enhances nucleation, gel formation, and pore refinement, leading to significant microstructural densification. However, beyond a critical threshold, further addition results in particle agglomeration, reduced effective surface area, and the formation of defects such as secondary porosity and weak zones. Consequently, the concept of a single “optimal” NS dosage is not universally applicable. Instead, the reported variations should be interpreted as system-specific optima that depend on the balance between chemical reactivity and physical dispersion. Therefore, the design of NS-modified geopolymers should be based on a holistic approach that considers not only the NS content itself, but also the entire mixture composition and processing conditions.

A selection of representative studies related to the above-mentioned factors is summarized in Table 2, highlighting the types, properties, and dosage ranges of NS used in geopolymer systems.

As well as those previously mentioned, the other factors that will influence the dosage of NS can also be found in the literature. One of these factors is the type of NS. In the case of colloidal NS, the mechanism of dispersion is a little bit more complicated than for dry powder. Guo et al. [67] show that dispersion is most effective in the presence of additional components and includes several mechanisms [67]. It initiates a controlled sol–gel transition, forming a continuous load-bearing Si–O–Si network, while partially soluble CaSO_4_ and CaCO_3_ particles are strongly anchored to the gel and effectively transfer stresses. At the same time, colloidal SiO_2_ and secondary Al(OH)_3_ fill nanometer-scale pores (~8 nm), markedly densifying the structure. The incorporation of a silane coupling agent further creates an organic–inorganic hybrid network, which enhances toughness and hardness without compromising strength [67].

In turn, Yang et al. [56] investigated different conditions of silica catalysis. Alkali-catalyzed NS consists of well-dispersed, highly reactive spherical SiO_2_ nanoparticles that promote uniform geopolymer gel formation, whereas acid-catalyzed NS tends to agglomerate due to less uniform morphology, thereby limiting its effective interaction with the geopolymer matrix [56]. The two types of NS influence the geopolymer gel structure in distinctly different ways, primarily due to differences in dispersion and surface reactivity [56,68]:

- Alkali-catalyzed NS exhibits superior dispersion within the alkaline geopolymer environment, which enhances its role as both a reactive silica source and a nucleation site. This promotes the simultaneous and homogeneous formation of C-(N)-A-S-H and N-A-S-H gels, leading to a dense, continuous, and highly uniform gel network. As a result, pore size is refined, nanoscale heterogeneity is reduced, and gel connectivity is significantly improved.
- Acid-catalyzed NS shows limited dispersion and a tendency to form agglomerates, especially at higher dosages. While it still contributes additional reactive silica and partially refines the geopolymer microstructure, the gel formation is more localized and less uniform. This results in a discontinuous gel network with increased structural heterogeneity and a higher likelihood of residual pores or weak interfacial zones.

Overall, alkali-catalyzed NS is markedly more effective in tailoring the geopolymer gel structure, enabling uniform gel growth and improved nanoscale continuity, whereas acid-catalyzed NS provides only moderate benefits due to dispersion-related limitations [56]. Similar results were obtained by Chen et al. [68], who also synthesized colloidal NS sols via the sol–gel method, produced under acid-catalyzed (AcC-NS) and alkaline-catalyzed (AlC-NS) conditions [68].

The other important factor is also the temperature of the curing process. Studies provided by Seenipeyathevar et al. [69] show that room-temperature curing provides performance comparable to, and in many cases better than, hot curing at 60 °C for geopolymer ferrocement systems. In particular, an optimized mix containing ≈80% GGBS and NS achieved a strength increase of up to ~329% under ambient curing, compared to ~143% under hot curing, while the addition of NS (≈1.5–2%) further enhanced strength by ~114% at room temperature versus ~91% at 60 °C [69]. The combined use of GGBS and NS accelerates geopolymerization and promotes early strength development, enabling dense microstructure formation without external heat input; as a result, the need for energy-intensive hot curing can be eliminated, making such systems more practical, economical, and sustainable for real structural applications [69]. Similar results were obtained by Raja and Sujatha [70]. They confirmed that geopolymer concrete based on GGBS exhibited very good mechanical properties without the need for heat curing, which significantly enhances its potential for in situ applications and reduces the energy demand of the production process [70].

Similar results connected with the application of the temperature were achieved by Zidi et al. [23], demonstrating that the effectiveness of NS strongly depends on the curing temperature, as the incorporation of 5% NS leads to significant strength enhancement at both 20 °C and 80 °C. Notably, after 28 days, the relative increase in compressive strength due to NS is more pronounced at 20 °C (about 57%) than at 80 °C (about 41%), indicating that NS efficiently compensates for the slower geopolymerization under ambient curing conditions [23]. This behavior is attributed to the acceleration of reaction kinetics and microstructural densification induced by NS, which makes it possible to achieve high performance without the need for elevated-temperature curing [23].

Alternatively, it is also possible to apply microwave curing for the geopolymers reinforced by NS. The research shows that this method actively accelerates and intensifies geopolymerization, enabling the achievement of high mechanical properties with significantly reduced processing time and energy consumption [71].

Overall, there is no universal “optimal” dosage of NS, as the effective range strongly depends on the precursor type, CaO content, NaOH molarity, fiber reinforcement, and the nanoparticle dispersion method (e.g., ultrasonication or in situ growth) [5]. Additional influence on the optimization of NS has simultaneous control of the alkali-to-binder ratio and the water-to-binder ratio [12]. These parameters govern geopolymerization kinetics, particle packing, and the risk of nanoparticle agglomeration. Nevertheless, most studies consistently report an effective NS dosage in the range of 0.5–2.0 wt.% of the binder [5].

Moreover, the results of some research indicate that mechanical and durability-related optima do not coincide, as the maximum strength is achieved at a lower NS dosage, whereas the minimum water absorption and highest resistance to sulfate attack occur at slightly higher dosages [72]. This divergence reflects the competing roles of NS, where moderate contents enhance nucleation and load transfer, while higher contents further densify the pore structure but begin to promote particle agglomeration that limits strength gains [72].

### 4.3. Influence on Fresh Properties of Geopolymers

**Figure 4:**
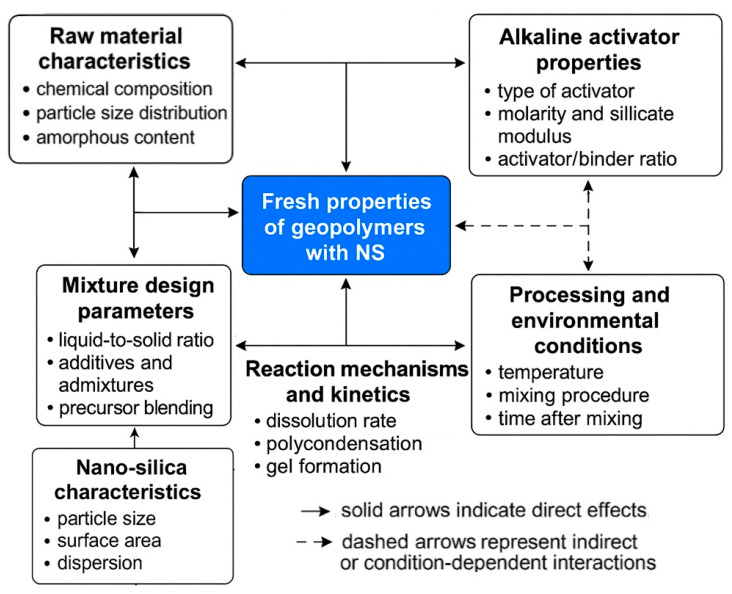
Conceptual framework illustrating the interrelated factors influencing the fresh properties of geopolymers with NS.

The fresh properties of geopolymers play a crucial role in determining their processing and performance, and are strongly affected by mixture composition, activator characteristics, and curing conditions. It should be emphasized that the influence of individual parameters on the fresh properties of geopolymers is highly interdependent and non-linear, resulting from complex interactions between material composition, chemical reactions, and processing conditions. The most important parameters that influence fresh properties are indicated in Figure 4.

The investigation provided by Khal et al. [64] shows that the incorporation of NS significantly reduces the setting time of geopolymer concrete due to its extremely high specific surface area and strong pozzolanic reactivity, which accelerate dissolution and geopolymerization reactions. At the same time, NS alters the workability of fresh mixtures by increasing water demand and particle flocculation, often necessitating the use of superplasticizers to maintain adequate flowability [64]. However, this behavior differs fundamentally from that observed in conventional OPC-based systems modified with pozzolanic materials. In OPC systems, pozzolanic additives typically increase the setting time because their reactivity is secondary and depends on the prior formation of Ca(OH)_2_ during cement hydration. The pozzolanic reaction proceeds relatively slowly and contributes mainly to later-age strength development. In contrast, geopolymer systems are governed by a different chemical mechanism, where setting is controlled by the dissolution of aluminosilicate precursors and the polycondensation of Si–O–Al species into a three-dimensional network. In this context, silica is a primary reactant rather than a secondary pozzolanic component. NS accelerates this process through (i) the rapid supply of highly reactive silica species, (ii) the provision of a high density of heterogeneous nucleation sites for N-A-S-H and C-(N)-A-S-H gel formation, and (iii) the enhancement of dissolution–polycondensation kinetics. As a result, a continuous gel network is formed more rapidly, leading to a reduction in setting time [64].

Nevertheless, this effect is strongly system-dependent. In some cases, especially at higher NS dosages or in silica-rich systems, an increase in setting time has been reported [64]. This delay is attributed to the formation of excess silicate oligomers, which require a longer time for reorganization and polycondensation into a stable geopolymer network. Therefore, the influence of NS on the setting time in geopolymers should be understood as a balance between kinetic acceleration (nucleation and dissolution) and structural reorganization processes, rather than as a direct analogy to pozzolanic reactions in OPC systems.

The above effects highlight the dual role of NS as both a reactivity enhancer and a rheology-controlling additive in geopolymer systems [64]. When incorporated into fly ash- and GGBFS-based geopolymer mortars, NS accelerates the setting time due to its high surface reactivity and nucleation effect, while simultaneously reducing workability (slump and flow) as a result of increased water demand and mix viscosity [73]. The addition of NS also increases viscosity and yield stress while introducing pronounced thixotropic behavior associated with time-dependent rebuilding of the flocculated structure [74].

This behavior was also confirmed by Mortada et al. [75]. NS accelerates the geopolymerization process, as evidenced by a reduced setting time and faster heat release during the reaction [75]. This effect is attributed to its high specific surface area, its ability to fill fine pores within the geopolymer matrix, and the increased number of nucleation sites for reaction products. Notably, the addition of 1 wt.% NS shortened the setting time by approximately 35%, from about 20 to 13 min [75].

Other results were presented by Ahmed et al. [76]. In their work, the addition of NS in the GGBFS-based geopolymer paste did not always shorten the setting time. The study provided by Ahmed et al. [76] resulted in prolonged initial and final setting times, with the delay increasing as the NS dosage increased [76]. The authors explain that the prolonged setting time is due to the increased availability of reactive silica introduced by NS, which leads to the formation of more silicate oligomers and consequently requires a longer time for their polycondensation into a stable geopolymer network [76].

Excessive NS content also leads to particle agglomeration, which disrupts uniform dispersion and results in secondary porosity, counteracting the intended densification of the geopolymer matrix. Moreover, due to its high surface area and surface energy, surplus NS absorbs mixing water and consumes reactive Ca^2+^, which can inhibit the hydration and activation of blast furnace slag and fly ash, ultimately reducing strength development and durability [77]. This phenomenon is also confirmed by Tian et al. [12]. They show that nanomaterials generally reduce the workability of self-compacting geopolymer concrete (SCGC), with the adverse effect being more pronounced at low water-to-binder ratios. The alkali content shows a non-linear influence: increasing alkali-to-binder ratio from 4.0% to 4.4% decreases the slump flow, while a further increase to 5.0% leads to a renewed improvement in flowability [12]. Additionally, they confirms than NS reduces the slump flow to a greater extent than other nano-additives, for example, nano-calcium carbonate when used at the same dosage [12].

Shumuye et al. [5] suggest that the influence of NS on workability depends more on dispersion and mixture composition than on the mere presence of the nanoparticle [5]. Based on that, Yi et al. [78] proposed a solution against deterioration in workability based on improvement dispersion [78]. They introduced NS in the form of a water-in-air Pickering emulsion (“dry water”), thanks to which they effectively eliminated the trade-off typically associated with nano-additives between enhanced mechanical strength and deteriorated rheological properties of geopolymers [78]. In this form, NS induces a coarsening effect, whereby particles transition from the nanoscale to the microscale, while simultaneously acting as a ball-bearing system that reduces internal friction among solid particles [78]. As a result, a reduction in viscosity and yield stress is observed despite the presence of a nano-additive, accompanied by an increased degree of geopolymerization and improved strength. By contrast, the direct addition of NS leads to increased viscosity and yield stress due to the higher specific surface area and water demand, confirming the superiority of the dry-water concept as an effective strategy for the balanced design of rheological and mechanical properties in geopolymers [78].

Another solution was proposed by Murugesan et al. [79]. They examined a hybrid nano-engineered geopolymer composite containing NS (≈5 wt.% of binder), graphene oxide, and CNTs, focusing not only on hardened performance but also on the fresh-state behavior during mixing and casting. It was observed that ultrasonication combined with mechanical milling enabled good dispersion of NS, allowing workable mixes to be achieved at low water-to-binder ratios of 0.30–0.35 without visible segregation or excessive stiffening [79]. As a result, despite the high nano-additive content, the fresh composite maintained adequate workability for molding while enabling rapid geopolymerization and early strength [79].

The other influence of NS on fresh properties is connected with the reduction in dry shrinkage. Xu et al. [80] showed that sol-type NS synthesized via the sol–gel method was shown to effectively mitigate drying shrinkage in fly ash–slag geopolymers [80]. At dosages ≤ 0.4%—with the strongest effect at 0.2%, reducing shrinkage by ~14.5%—this behavior was attributed to pore structure refinement, disruption of capillary continuity, and reduced water evaporation, resulting from a denser and less connected pore network [80].

In turn, Xia et al. [81] demonstrate that combining superabsorbent polymers with NS is a highly effective strategy for designing low-shrinkage geopolymer concrete without compromising strength, as NS acts as a compensating material that offsets the strength loss induced by superabsorbent polymers [81]. As a result, the superabsorbent polymers–NS system brings geopolymer concrete closer to practical structural applications, with direct relevance to precast elements, massive concrete components, and next-generation low-carbon concretes [81].

The understanding and control of fresh properties in geopolymers incorporating NS are particularly critical when these materials are intended for extrusion-based additive manufacturing. In 3D printing, requirements related to rheology, buildability, and structural stability impose additional constraints that go beyond conventional casting applications. Moreover, the research shows that the behavior is not always in line with previous research. In contrast to conventional applications where NS acts as an active reaction accelerator, in 3D printing applications, NS primarily functions as a regulator of hardening kinetics and rheological behavior, rather than an initiator of rapid geopolymerization. NS does not excessively accelerate setting—which would be detrimental for 3D printing—but instead allows precise tuning of the static yield stress (τ_s_) development and compensates for the reduced alkalinity introduced by sodium carbonate activation. As a result, the material maintains sufficient structural buildup while preserving an extended printable time [82]. Regarding rheology and printability, NS significantly enhances thixotropy, enabling the material to rapidly rebuild its structure after shear during extrusion, and improves filament shape stability through a slower reduction in the shape retention ratio. Extrudability is improved without compromising pumpability; however, increasing the NS dosage raises τ_s_ and consequently shortens the printable time. This behavior indicates that NS acts as a fine-tuning rheological regulator: insufficient amounts lead to overly soft mixtures, while excessive dosages cause a premature loss of printability [82].

Despite the refinement of the microstructure, NS alone does not eliminate shrinkage-induced cracking in 3D-printed filaments, which is attributed to rapid setting and pronounced chemical shrinkage [83]. Therefore, the effective mitigation of shrinkage cracking requires synergistic use of NS with complementary additives, such as methyl cellulose or calcium aluminate cement, that regulate moisture retention, reaction kinetics, and early-age dimensional stability [83].

It should be emphasized that, despite numerous studies on NS-modified geopolymers, there is currently no universal methodology that allows the dosage of NS to be determined a priori. The optimal NS content remains strongly system- and application-dependent, and must typically be established through experimental optimization. Variations in precursor chemistry, activator composition, dispersion efficiency, and curing conditions significantly influence the effectiveness of NS. In addition, different application targets—such as mechanical performance, durability, or rheological behavior—may require different optimal dosage ranges. Therefore, NS dosage should be treated as an iterative design parameter rather than a fixed or universally defined value.

## 5. Mechanisms of Nano-Silica Action in Geopolymers

NS affects geopolymer systems through a complex hierarchy of chemical, kinetic, and microstructural mechanisms, which collectively govern their fresh-state behavior, mechanical performance, and long-term durability. Xu et al. [72] identified five main mechanisms by which NS acts:

- The filling effect: NS particles fill micro- and nanopores in the geopolymer matrix and the ITZ, leading to a denser microstructure and reduced porosity [35,84,85].
- The particle size effect: smaller NS particles provide a higher specific surface area, enhancing reactivity, pore refinement, and interaction with reaction products [35,84].
- The nucleation effect: NS acts as heterogeneous nucleation sites for the precipitation of reaction gels (e.g., N-A-S-H, C-A-S-H), accelerating geopolymerization and early-age strength development [35,85,86].
- The microcrack bridging effect: NS particles and the associated dense gel network bridge microcracks, limiting their opening and delaying crack propagation under load [35,85].
- The crack deflection effect: when a crack encounters NS-reinforced regions, its path is deflected or branched, increasing fracture energy and improving toughness and durability [35].

The above effects are the most commonly described in the literature as an explanation of basic phenomena connected with NS-reinforced geopolymers. However, in the literature, some other mechanisms are also described. The basic one is the reactive silica effect. It refers to the role of NS as a source of highly reactive amorphous Si, which enhances dissolution–polycondensation reactions and increases the degree of network polymerization through the formation of longer and more interconnected silicate chains [87,88]. The influence of NS on the reaction degree and structure was examined using reactive molecular dynamics by quantifying hydroxyl consumption and the fraction of bridging oxygen atoms in the N-A-S-H network. Compared to neat N-A-S-H gels, NS increased the reaction degree by about 10% and the network complexity by roughly 6%, with the effect being most pronounced at low Si/Al = 1.5. This enhancement was attributed to NS providing additional nucleation sites and reactive silicon species, which promote the formation of a more continuous and three-dimensional aluminosilicate network [18].

On the next level, the important thing is the early-age acceleration effect. It arises from the high density of nucleation sites introduced by NS, which promotes rapid formation of the load-bearing gel network and results in a pronounced increase in early-age strength [52]. The important role also includes the pore refinement effect. It refers to the ability of NS to shift the pore size distribution from larger capillary pores toward finer gel pores, leading to reduced permeability and a significant limitation of ionic transport through the geopolymer matrix [52]. NS exhibits a pronounced pozzolanic effect by reacting with free Ca^2+^ ions and alumina species released from slag and fly ash under alkaline activation, leading to the formation of additional binding phases such as C-S-H, C-A-H, and C-A-S-H gels. These secondary reaction products fill capillary and gel pores, refine the pore structure, and strengthen the geopolymer matrix at the microstructural level. As a result, the matrix becomes denser and more chemically stable, which directly contributes to enhanced compressive strength and durability [77].

The other effect described in the literature is the fracture energy enhancement effect, which arises because NS promotes crack deflection, branching, and delayed crack propagation, thereby increasing the energy required for crack growth and imparting a higher degree of quasi-plastic behavior to geopolymer composites [88,89]. In the case of composites with fibers or recycled aggregates, a crucial role is played by the ITZ densification effect (interface improvement). The ITZ densification effect results from the preferential accumulation of reaction products around NS particles, leading to a denser and mechanically stronger interfacial transition zone that is no longer the weakest component of the composite [52,84,85].

The effect that significantly influences the performance of geopolymers with NS is the alkali immobilization effect, which arises from the increased density and cross-linking of NS-modified gels. It promotes the incorporation and retention of Na^+^ and K^+^ ions within the geopolymer network, thereby reducing alkali leaching and mitigating efflorescence [88,90]. A similar mechanism can also be observed in the ionic shielding effect. The ionic shielding (barrier) effect results from NS-induced densification of the gel nanostructure, which restricts the transport of water and aggressive ions such as Cl^−^ and SO_4_^2−^, thereby enhancing chemical resistance and environmental durability [23,88].

**Figure 5:**
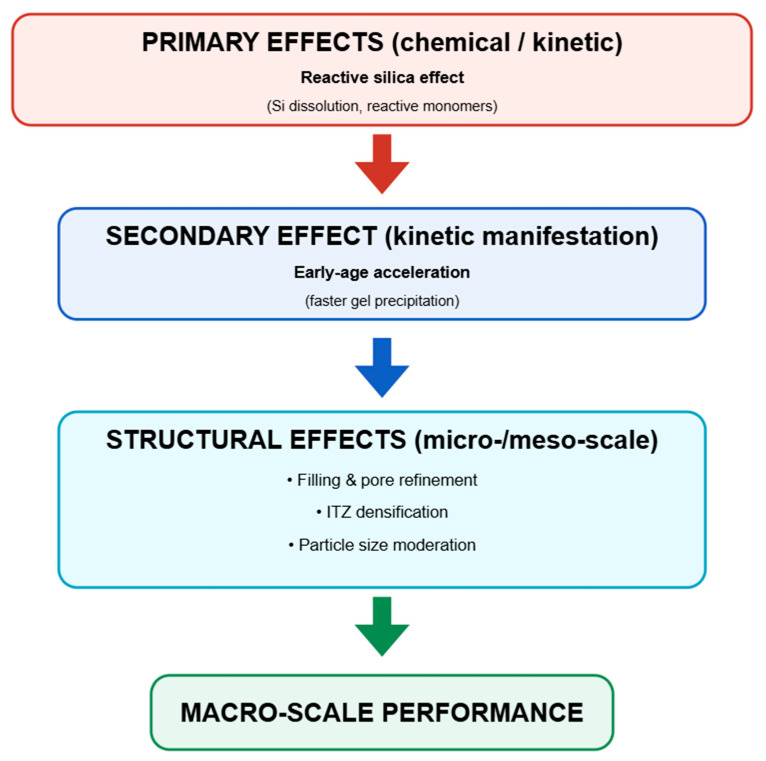
Conceptual representation of the multi-scale and cause-and-effect mechanisms governing the influence of NS on geopolymer systems.

To clarify the causal hierarchy and multi-scale interactions underlying the influence of NS on geopolymer systems, the overall mechanism is conceptually summarized in Figure 5.

NS’s influence on geopolymer systems can be presented through a hierarchical cascade of mechanisms (Figure 5), beginning with primary effects that act directly at the chemical and kinetic level of geopolymerization. At this fundamental stage, NS serves as a source of highly reactive amorphous silica (reactive silica effect), directly modifying the composition and degree of polymerization of the formed gels, while simultaneously providing heterogeneous nucleation sites that control the rate and spatial distribution of reaction products (nucleation effect); these two mechanisms represent the true driving forces of the system. As a direct consequence, secondary effects emerge, most notably the acceleration of early-age reaction kinetics, which is not an independent process but rather the manifested outcome of increased nucleation density and enhanced silica availability, observable macroscopically as faster strength development and earlier structural densification. These processes further give rise to micro- and mesoscale structural effects, including pore filling by NS and gels, refinement of pore size distribution from capillary to gel pores, densification of the interfacial transition zone, and particle size-related modifications that may either enhance or attenuate performance depending on dispersion quality. Ultimately, the cumulative result of this cascade is reflected at the macroscopic scale through improved mechanical performance and durability, manifested by microcrack bridging and deflection, increased fracture energy, immobilization of alkali ions, and the formation of a refined barrier microstructure limiting the transport of aggressive species.

During the experiments, the authors pay attention to other aspects of this mechanism. Table 3 summarizes selected, practically relevant aspects of NS interactions with geopolymer microstructure as reported in the literature. Emphasis is placed on integrating chemical, microstructural, and performance-related perspectives by identifying the dominant mechanisms operating at different length scales and the key techniques used for their characterization. This overview is not intended to be exhaustive, but rather to highlight the most commonly reported and application-relevant mechanisms.

In Table 3, the effects of NS on geopolymer materials are analyzed across three characteristic length scales: nano-, micro-, and mesoscale. The nanoscale (≈1–100 nm) refers to the level of chemical reactions and gel structure, where NS influences geopolymerization kinetics and the chemistry of the binding gel. The microscale (≈0.1–100 µm) describes the development of the microstructure, including pore system refinement and densification of the interfacial transition zone (ITZ). The mesoscale (≈0.1–10 mm) represents the structural level at which these microstructural modifications translate into macroscopic mechanical behavior and overall material performance.

It is also worth noting that NS can have a synergistic effect with other components. In multi-additive compositions, NS can play additional roles. The results obtained by Cheng and Tang [91] confirm that precise control of matrix composition and the compatibility among nano-additives play a more decisive role in performance enhancement than the mere addition of a single NS component. In combination with graphite nanoparticles and nickel-plated multiwalled carbon nanotube fibers, silica fume promotes densification of the cementitious/geopolymer matrix, stabilizes the interfacial transition zone (ITZ), and provides a favorable environment for the effective anchoring of carbon nanotubes and graphite nanoparticles within the matrix [91]. Similarly, Vignesh et al. [54] pointed out that NS does not act effectively as a stand-alone additive; instead, it achieves its maximum effectiveness in well-designed, compatible nano–fiber systems, where control of matrix composition, dispersion, and interfacial characteristics is more critical than the mere presence of NS itself [54].

## 6. Influence of Nano-Silica on Porosity and Pore Structure

NS has been investigated as an effective modifier of geopolymer matrices due to its ability to alter pore structure development across multiple length scales. NS influences total porosity, pore size distribution, connectivity, and pore network characteristics, with particular emphasis on transport- and durability-related implications. Basically, NS acts as an efficient nano-filler by occupying fine voids within the geopolymer matrix, thereby reducing the total pore volume and increasing matrix compactness [60]. As a result, NS significantly shifts the pore size distribution toward micro- and mesopores, limiting the presence of harmful macropores and enhancing the mechanical performance and durability of the composite [60]. It effectively suppresses pore coarsening, particularly in the range of pores larger than 1 µm [92]. Since these large capillary pores are most detrimental to both mechanical strength and thermal transport, their reduction leads to a denser microstructure and improved structural and heat-transfer performance [92].

**Figure 6:**
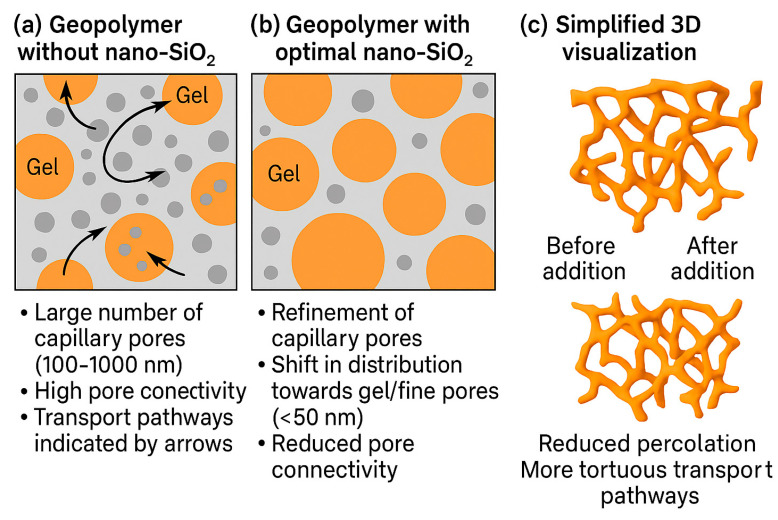
Schematic illustration of the effects of NS on porosity and pore structure of geopolymer matrices: (**a**) capillary-dominated pore system with high connectivity and efficient transport pathways, (**b**) refinement of pore size distribution and shift toward gel-scale porosity, accompanied by reduced pore connectivity after NS addition, and (**c**) conceptual three-dimensional visualization of pore networks highlighting increased tortuosity and transport-related performance improvements. The figure is a conceptual representation developed by the authors based on trends reported in the literature.

To better illustrate the general mechanisms governing pore structure evolution in NS-modified geopolymers, a conceptual schematic is presented in Figure 6. This figure does not represent a single experimental dataset, but rather summarizes commonly reported trends in the literature, including pore refinement, reduced connectivity, and increased tortuosity of the pore network resulting from NS addition.

The practical confirmation of these mechanisms can be found in the research made by Chen et al. [93]. NS with an average particle size of approximately 20 nm and a specific surface area of about 240 m^2^/g was incorporated at dosages ranging from 0 to 2.5 wt.% of total binder (coal gangue and GGBF slag). The reference material exhibited a total porosity of approximately 4.90%, while the incorporation of 1 wt.% NS reduced porosity to about 2.16%, corresponding to a reduction of roughly 56% [93]. Pores were classified according to equivalent diameters in the micrometer range (0–50 µm, 50–100 µm, 100–150 µm and >150 µm), and no nanoscale critical pore diameter was defined. Image-based pore distribution analysis and SEM observations indicated reduced pore interconnection and fewer defects at optimal NS contents (approximately 1–1.5 wt.%) [93].

In mortar systems containing red mud-based geopolymer hollow microspheres serving as phase-change material carriers, NS applied in the form of a surface-grafted SiO_2_ coating deposited via a sol–gel process (TEOS hydrolysis), rather than as a dispersed nano-additive. The matrix itself consisted of ordinary Portland cement, while the microsphere precursor was a blend of red mud and GGBFS. Mercury intrusion porosimetry performed after 28 days of curing showed a total porosity of approximately 12.1% for the control mortar, increasing to around 16.2% with the addition of 20% uncoated PCM microspheres. The application of NS coating limited this increase, resulting in a porosity of approximately 15.5%. The critical pore size remained in a narrow range of about 38–42 nm for all systems, indicating that the NS coating did not shift the critical pore diameter but modified the pore population distribution. Qualitative evidence of reduced pore connectivity was provided by the suppression of pore coarsening, a pronounced reduction in pores larger than 1 µm [92].

A different behavior was reported for a geopolymer synthesized from simulated lunar soil (TJ-1 lunar soil simulant), representing a low-reactivity silica–alumina precursor system [57]. Colloidal NS with an average particle size of approximately 30 nm was introduced as an aqueous dispersion at dosages of 0.50, 0.75, 1.00 and 1.25 wt.%, with 0.75 wt.% identified as the optimal content. The reference material without NS exhibited a total porosity of 40.09%, while NS addition reduced porosity to 35.21% at 0.50 wt.% and 35.57% at 0.75 wt.% [57]. At the highest dosage (1.25 wt.%), porosity increased to 37.42%, indicating a partial loss of the densification effect. The reference system was characterized by dominant capillary pores in the range of approximately 100–300 nm. The addition of NS suppressed coarse pores larger than 300 nm, whereas excessive NS content reintroduced larger pores due to agglomeration. Pore connectivity was qualitatively reduced by NS through sealing of through-cracks and limitation of interconnected coarse pores [57].

NS also influences the porosity in the case of high-temperature application. In the MK-based geopolymer system, NS governs early mesopore refinement (~5–10 nm), while high-temperature exposure induces macropore development followed by pore collapse and densification [42]. MIP revealed a total porosity of 15.98% for the geopolymer mortar cured at room temperature. After thermal exposure, porosity increased to 30.43% at 400 °C, while further heating to 1000 °C resulted in a reduction to 16.18%, attributed to viscous sintering effects. At ambient conditions, the pore structure was dominated by mesopores centered around approximately 6.2 nm, whereas after exposure to 1000 °C, macropores in the range of roughly 2800–6700 nm became dominant, depending on aggregate gradation. Qualitative analysis indicated a reduction in pore connectivity at high temperatures due to pore coalescence, followed by viscous sintering and pore closure [42]. The observed porosity minimum at 1000 °C confirms that silica-rich binders promote viscous sintering rather than continuous pore growth [42].

The incorporation of NS has a pronounced and consistent effect on the pore structure of geopolymer matrices, acting across multiple length scales to refine porosity and enhance material compactness. NS modifies both the total pore volume and pore size distribution, predominantly shifting the system from capillary-dominated porosity toward a finer gel-pore structure. The primary mechanisms responsible for these changes include the nano-filler effect, which enables NS to occupy micro- and nanopores, and the nucleation effect, which promotes the formation of additional geopolymer gel phases. As a result, NS reduces pore connectivity and increases tortuosity of the pore network, effectively limiting fluid transport and ion ingress. Importantly, the influence of NS on porosity is strongly dosage-dependent. At optimal contents, NS significantly decreases total porosity and suppresses the formation of harmful large pores, leading to a denser and more homogeneous microstructure. However, excessive NS addition may result in particle agglomeration, secondary porosity, and a partial loss of the densification effect.

Overall, NS enables controlled tailoring of the geopolymer pore structure, which directly translates into improved durability, reduced permeability, and enhanced resistance to aggressive environments. The effectiveness of this modification is governed by a balance between dispersion quality, chemical reactivity, and mixture composition, highlighting the need for system-specific optimization.

## 7. Nano-Silica and Interfacial Transition Zone (ITZ) Optimization

ITZ is classically defined as a distinct microstructural region located between the bulk binding matrix and inclusions such as aggregates or fibers [94,95]. In conventional cementitious materials, the ITZ is characterized by higher porosity, a disturbed phase assemblage, and inferior mechanical properties compared to the bulk matrix. However, there are some differences between the ITZ in OPC and geopolymers. In geopolymers, the ITZ is significantly stronger than in OPC concrete, as it consists of a continuous, dense gel layer rich in N-A-S-H rather than a porous zone with large CH and ettringite crystals [96]. Nanoindentation results show that the elastic modulus and hardness of the geopolymer ITZ are comparable to, or even higher than, those of the bulk paste, whereas the OPC ITZ remains a mechanically weaker region despite locally hard crystalline phases [96]. Moreover, the geopolymer ITZ exhibits spatial heterogeneity, with the top and bottom ITZs showing superior mechanical properties compared to the lateral ITZ due to local densification, fly ash distribution, and sedimentation effects [96]. NS enhances aggregate–matrix bonding by densifying the interfacial transition zone, where its fine particles fill micro-pores and promote the formation of a continuous, gel-rich microstructure [97,98]. As a result, the ITZ becomes more compact and homogeneous, reducing stress concentrations and improving load transfer between the aggregate and the geopolymer matrix [97]. The main differences between the ITZ in OPC and geopolymers are pointed out in Table 4.

The ITZ is critical in nano-modified composites because it governs crack initiation, ion and moisture transport, and interfacial load transfer. In nanocomposites, improvements in strength, durability, and interlayer adhesion—particularly relevant for 3D-printed systems—are predominantly realized through the deliberate engineering of the ITZ, where the material’s overall performance advantage is effectively determined [99]. In contrast to traditional mineral additives, NS does not merely reside adjacent to the ITZ but actively contributes to its formation. NS particles become an inherent part of the interfacial region, acting as preferential sites for heterogeneous nucleation of geopolymer gels, as reactive constituents of the binding phase, and as a chemical “glue” enhancing interfacial bonding between phases. Consequently, the ITZ formed in nano-modified geopolymers is not an incidental by-product of processing but a deliberately designed zone with tailored chemistry, microstructure, and load-transfer capability [100]. This mechanism—formation of the ITZ at the NS–N-A-S-H interface—was investigated by Gual et al. [18] using reactive molecular dynamics simulations (ReaxFF), which allow chemical reactions and atomic diffusion to be tracked explicitly. The ITZ thickness (~10 Å) and its higher density, increased structural order, and reduced ion mobility were identified through density profiles, radial distribution function analysis, and mean square displacement calculations [18]. The key mechanisms connected with the ITZ in geopolymers with NS are:

- Heterogeneous nucleation—NS promotes heterogeneous nucleation of N-A-S-H and C-(N)-A-S-H gels at aggregate or reinforcement surfaces, leading to localized matrix densification and the formation of an ITZ with mechanical properties comparable to or exceeding those of the bulk matrix, contrary to the classical view of the ITZ as the weakest phase [7,100].
- Nano-filler effect—NS particles fill micro- and mesopores within the aggregate–matrix interfacial region, disrupting capillary pore connectivity and reducing local porosity such that the ITZ no longer represents the most porous zone, thereby enhancing impermeability and durability [98,101].
- Chemical effects specific to geopolymer systems—in geopolymer matrices, NS modifies the local Si/Al ratio and enhances gel polymerization and cross-linking, producing a chemically distinct and stabilized ITZ that is deliberately engineered to improve interfacial bonding and stress-transfer efficiency [7,90].

**Figure 7:**
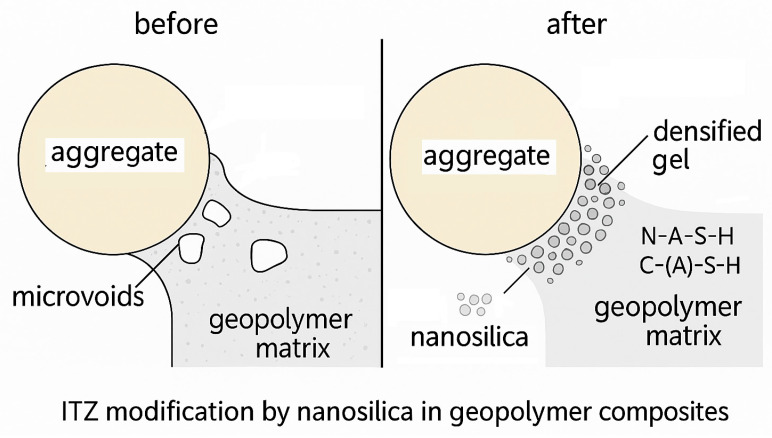
Schematic illustration of ITZ modification by NS in geopolymer with aggregate.

Figure 7 shows a conceptual schematic illustrating the mechanisms by which NS modifies the ITZ in geopolymer composites through physical densification and chemical interaction at the aggregate–matrix interface.

The indirect confirmation of the presented mechanisms is visible with the increase in mechanical properties. Guan et al. [18] evaluated the mechanical properties using uniaxial tensile simulations based on reactive molecular dynamics, allowing stress–strain behavior of neat N-A-S-H, NS-modified N-A-S-H, and the ITZ to be directly compared. The results showed that tensile strength increased by 10–40% with NS addition, reaching a maximum of ~4.1 GPa at Si/Al = 2.5–3.0, and revealed a clear strength hierarchy of ITZ > silica-modified N-A-S-H > neat N-A-S-H, demonstrating that the ITZ is the strongest component rather than a weak zone [18].

The particular meaning this mechanism has lies in the case of using recycled aggregates. Yuan et al. [37] addressed the problem of the poor mechanical performance of geopolymer concrete containing recycled aggregates, caused by a weak and highly porous ITZ associated with adhered old mortar. The research investigated an in situ NS generation method using tannic acid and sodium silicate to modify recycled aggregates and strengthen all ITZs in the composite [37]. The results showed that NS is crucial for refining the pore nanostructure, enhancing chemical bonding and nucleation at interfaces, and significantly improving mechanical strength, making recycled-aggregate geopolymer concrete more viable for structural applications [37]. Similarly, Chen et al. [68] demonstrate that alkaline-catalyzed NS fundamentally modifies the OPC–geopolymer concrete ITZ, transforming it from a porous, adhesion-dominated region into a dense, reactive, and load-bearing layer capable of efficient stress transfer, which is directly relevant to repair and prefabrication applications. At the microstructural level, NS fills micro-pores and promotes the formation of a continuous and compact transition zone, as confirmed by SEM observations showing gel bridging between OPC and geopolymer matrices [68]. EDS line-scan analyses further reveal a gradual decrease in Ca content from the OPC side and a corresponding increase in Si and Al toward the geopolymer side, indicating the formation of a continuous chemical gradient rather than a sharp interface [68]. Additionally, NS actively participates in interfacial reactions, where Ca(OH)_2_ from OPC reacts with SiO_2_ to form additional C-S-H alongside coexisting C-(A)-S-H and N-A-S-H gels, as corroborated by FTIR peak shifts and intensification of Si–O–T bonds, confirming that the ITZ becomes chemically active rather than inert [68].

Analogously, NS has an influence on the ITZ between the matrix and fibers. Wu et al. [102] performed SEM observations of the fiber–matrix contact zones, which can be regarded as an indirect characterization of the ITZ, covering the steel fiber–geopolymer interface, the polypropylene fiber–geopolymer interface, and locally densified matrix regions associated with the presence of NS. The microstructural analysis revealed reduced interfacial gaps, frictional marks and mechanical anchoring of fibers, as well as a more compact matrix surrounding the fibers, indicating an improved fiber–matrix interaction enabled by NS [102]. Also, Vignesh et al. [54] emphasize that the most pronounced improvements in the ITZ are achieved in hybrid systems combining NS with fibers such as polyvinyl alcohol (PVA), steel, basalt, or carbon fibers. In these systems, NS enhances matrix–fiber adhesion, reduces defects and voids within the ITZ, and stabilizes the interface under cyclic loading and aggressive chemical exposure, acting as a form of “pre-emptive shielding” that protects the ITZ against stress concentration and degradation [54]. At the fiber–matrix ITZ involving steel fibers, NS increases the amount of geopolymer gel adhering to the fiber surface, thereby enhancing both chemical bonding and mechanical interlocking [103]. This improved interfacial adhesion enables more effective crack-bridging and load transfer by the steel fibers. However, excessive NS leads to particle agglomeration, increased local porosity, and a subsequent weakening of the fiber–matrix bond [103].

In the case of polymeric fibers, most of the research was connected with PVA. The provided research confirms that NS acts as an effective modifier of the fiber–matrix ITZ by densifying the geopolymer matrix through nano-filler and nucleation effects, reducing micro-pores and microcracks around PVA fibers, and enhancing fiber–matrix adhesion, which collectively improves stress transfer and promotes more efficient crack-bridging behavior [65]. The synergy between NS and PVA fibers yields significantly better results than using each additive separately, particularly in terms of resistance to freeze–thaw cycles and resistance to aggressive environmental conditions [104]. In the NS–PVA system, the addition of NS enhances the beneficial effects of PVA fibers on strength and durability while partially mitigating adverse effects associated with high PVA contents, such as increased porosity [105]. Zhang et al. [106] also define the role of NS to strengthen the interaction between the matrix and PVA as well as steel fibers. Effective fiber optimization is only achievable through nanoscale modification provided by NS, as the mechanisms are inherently synergistic [106].

The synergy effect was also confirmed between NS and polyolefin fibers, which arises from their complementary roles: polyolefin fibers primarily enhance impact resistance and ductility through crack-bridging, while NS densifies the matrix by refining the pore structure. Their combined use provides an optimal balance between load-bearing capacity and long-term durability in geopolymer concrete [107]. Dong et al. [108] also confirm that the presence of NS improves the dispersion of PE fibers and increases the number of fibers effectively aligned with the loading direction. At the same time, NS strengthens the fiber–matrix interfacial transition zone, which enhances fiber bridging capacity. As a result, higher bridging stresses and finer, more uniformly distributed cracks are achieved [108].

Moreover, in the case of the application of carbon fibers, NS functions as an active dispersing agent through adsorption onto the surface of fibers, thereby modifying their surface characteristics. This interaction enhances electrostatic repulsive forces between adjacent fibers, effectively suppressing fiber entanglement and mitigating agglomeration within the composite matrix [109].

Opara et al. [31] also show that colloidal NS is a highly effective additive for natural fiber-reinforced geopolymers. It enables the elimination of energy-intensive heat curing while achieving high load-bearing capacity and durability. Consequently, its use supports the development of prefabricated, low-carbon construction materials of the next generation [31]. Research with natural fibers was also provided by Assaedi et al. [110]. They show that flax fibers embedded in geopolymer matrices without NS undergo progressive degradation over time, including cracking, debonding, and a loss of structural integrity [110]. The addition of NS slows down this degradation process by modifying the geopolymer matrix, leading to better preservation of fiber continuity after long-term curing. NS contributes to a denser matrix and reduced alkalinity, which mitigates but does not completely prevent fiber deterioration. Importantly, the authors emphasize that NS limits fiber degradation rather than eliminating it entirely [110]. NS improves fiber–matrix interfacial bonding, leading to enhanced load transfer and crack-bridging efficiency. As a result, NS increases toughness and fracture toughness and may improve resistance to microcrack initiation and propagation [5].

This kind of phenomenon also has a place for other modern additives. Yu et al. [92] also observed a similar mechanism of an enhanced ITZ in PCM–geopolymer composites. The NS layer provides a highly reactive pozzolanic surface, promoting hydration products and ensuring intimate bonding between the PCM capsule and the cement matrix. As a result, the loose and porous ITZ typically observed around capsules without NS is effectively eliminated [92]. In this context the NS layer provides a highly reactive surface that can be described as pozzolanic in the background of OPC-based systems, where silica reacts with Ca(OH)_2_ to form secondary C–S–H phases [111]. However, it should be emphasized that the reactivity of NS differs from that of conventional pozzolans. In classical pozzolanic materials, reactivity is typically evaluated using standardized methods such as the Frattini test, Luxán conductivity test, or R^3^ test, which are based on portlandite consumption and long-term reaction kinetics. In contrast, nano-silica exhibits extremely fast reaction rates due to its very high specific surface area and amorphous structure, making these traditional methods less sensitive or not fully representative [111]. For NS, reactivity is more appropriately assessed using a combination of methods, including isothermal calorimetry (to capture acceleration of reaction kinetics), thermogravimetric analysis of Ca(OH)_2_ consumption (in OPC systems), electrical conductivity measurements, and advanced spectroscopic techniques such as NMR and FTIR, which provide insight into gel formation and polymerization. Moreover, in geopolymer systems, NS does not act solely as a pozzolanic additive, but rather as a primary reactive silica source and a nucleation agent that directly participates in geopolymerization. Therefore, its “pozzolanic activity” should be understood in a broader sense, encompassing both chemical reactivity and nucleation-driven acceleration of gel formation. In this context, the improved bonding observed at the PCM–matrix interface is attributed not only to classical pozzolanic reactions, but also to enhanced nucleation, rapid gel precipitation, and microstructural densification induced by NS [92].

## 8. Influence on Mechanical Performance

### 8.1. Mechanical Properties

Strength gains on the order of 7–49% are commonly reported in the case of the addition of NS to the geopolymer matrix, whereas enhancements exceeding ~60% are achievable but are typically associated with exceptionally effective nanoparticle dispersion [112]. NS enhances not only strength but also the fracture resistance of geopolymer concrete by significantly increasing both the fracture energy and the stress intensity factor. This indicates an improved ability to resist crack initiation and propagation, resulting in a less brittle and more damage-tolerant material [113]. Consequently, geopolymer concrete modified with NS exhibits superior fracture performance compared to conventional OPC concrete [113].

Jeevan et al. [61] demonstrate that NS plays a decisive role in enhancing the mechanical performance of geopolymer bricks by promoting microstructural densification and particle interlocking, as evidenced by an increase in compressive strength from 41.5 MPa to 45 MPa, tensile strength from 3.35 MPa to 4.5 MPa, and flexural strength from 6.2 MPa to 6.5 MPa compared to conventional geopolymer bricks [61]. These improvements are attributed to the incorporation of approximately 3 wt.% NS, which resulted in a denser and more uniformly interlocked geopolymer matrix [61]. Lakshmi et al. [62] confirm that the incorporation of 3 wt.% NS increased the compressive strength of geopolymer concrete by approximately 40%, while the splitting tensile and flexural strengths were enhanced by about 27% and 33%, respectively, compared to the reference mixture without NS [62].

The addition of NS to geopolymer mortars based on fly ash and GGBFS significantly enhances compressive strength, flexural strength, and bond strength by densifying the matrix, promoting N-A-S-H/C-A-S-H gel formation, and improving particle–matrix adhesion [73]. Also, NS led to a significant increase in the mechanical strength of geopolymer concrete based on a ternary geopolymer matrix composed of fly ash, GGBFS, and sugarcane bagasse ash (SBA) [114]. For the optimal mixture containing 4% NS, the highest compressive strength—approximately 41–43 MPa after 28 days of curing—and a splitting tensile strength of approximately 3.9 MPa were achieved, corresponding to an increase of about 25–30% compared to the reference sample without the NS additive. The authors attribute this improvement to microstructural densification and enhanced packing of the binding phases induced by the presence of NS [114]. Also, Hombali and Selvam [115] show that in the SBA-based geopolymer matrix (fly ash–GGBFS–sugarcane bagasse ash system), mechanical performance was evaluated in terms of compressive strength (CS), split tensile strength, and flexural strength. Compared with the control mix, the optimum mix containing 2.5 wt.% NS exhibited an increase of about 32% in compressive strength, while split tensile and flexural strengths increased by approximately 20–25%, which was attributed to matrix densification, improved ITZ quality, and accelerated geopolymerization; higher NS contents led to slightly lower strength gains due to agglomeration effects [115]. NS is also an effective modifier for ultra-high-strength geopolymetric concrete [116].

Moreover, geopolymers incorporating NS exhibit very high early-age strength development, reaching approximately 90% of their 28-day compressive strength within only 7 days. This rapid strength gain is achieved under ambient curing conditions, highlighting the strong accelerating effect of NS on geopolymerization kinetics [117]. Also, the early-age strength is improved by NS in lightweight geopolymer concrete by up to 25% already at the early stage of hardening [118]. NS cannot only enhance traditional solid geopolymers, but also others with increased porosity. In the research provided by Liang et al. [119], the incorporation of NS significantly enhances the mechanical integrity of porous geopolymer adsorbents, effectively mitigating their inherent brittleness and susceptibility to damage in aqueous environments [119]. An optimal NS content of approximately 2% results in an increase in compressive strength of about 63.5% compared to the NS-free material, primarily due to matrix densification and enhanced formation of geopolymer gels [119]. This strength improvement enables the porous geopolymer adsorbent to maintain structural stability during water exposure and repeated adsorption cycles [119].

It is also worth noting that NS, compared to other nanomaterials, shows better potential to improve mechanical properties. Shi et al. [120] compared NS, nano-calcium carbonate, and graphene nanoplatelets; among them, NS exhibited the strongest enhancement in mechanical properties in sludge-based geopolymers [120]. At an optimal dosage of 2% NS, the compressive strength increased by up to 108.2% and the flexural strength by 69.9% compared to the reference mixture. At the same doping level, NS was clearly more effective than nano-calcium carbonate and graphene nanoplatelets, highlighting its superior reactivity and compatibility with geopolymer gel phases [120]. Other research also suggests that NS improves macroscopic strength more effectively than nano-TiO_2_, despite the fact that NS itself exhibits lower intrinsic mechanical properties than TiO_2_ nanoparticles [19].

Table 5 summarizes representative results from the literature, highlighting the influence of the NS dosage, precursor type, and composite design on compressive, tensile, and flexural strength.

The data presented in Table 5 confirm that, although NS generally enhances mechanical performance, the magnitude of improvement varies significantly depending on system composition and dosage, reinforcing the non-universal and highly non-linear nature of its effect.

NS is a highly effective modifier of the mechanical performance of geopolymer composites, enabling significant improvements in compressive, tensile, and flexural strength, as well as fracture resistance. These enhancements are primarily attributed to microstructural densification, pore refinement, and improved interfacial bonding within the matrix. The key mechanism underlying strength enhancement is the acceleration of geopolymerization reactions, driven by the high reactivity and nucleation capability of NS. This leads to faster formation of a continuous and highly cross-linked gel network, resulting in increased early-age strength and improved load-bearing capacity. In addition, NS contributes to strengthening the interfacial transition zone (ITZ), enhancing stress transfer and reducing weak zones within the composite.

The improvements in mechanical properties are strongly dependent on NS dosage and dispersion. At optimal levels, NS promotes homogeneous gel formation and effective load transfer, while excessive addition leads to agglomeration, microstructural heterogeneity, and reduced mechanical efficiency. Furthermore, NS plays a critical role in advanced and hybrid systems, where it enhances the effectiveness of fiber reinforcement and synergistically improves toughness, fatigue resistance, and crack propagation control. In summary, NS acts not only as a strength-enhancing additive but as a key microstructural design tool, enabling the development of high-performance geopolymer materials with tailored mechanical behavior. However, its effectiveness is inherently system-dependent and requires careful optimization of composition and processing conditions.

### 8.2. Mechanical Properties at High Temperatures

Kanagaraj et al. [3] claim that NS exhibits consistent and well-documented benefits in geopolymer systems, where it enhances gel densification, refines pore structure, and improves post-fire mechanical performance. Its effectiveness is strongly temperature-dependent, transitioning from strength enhancement at ≤400 °C to damage mitigation and structural stabilization at higher temperatures [3]. However, other research also shows benefits from NS addition at much higher temperatures. Mansourghanaei and Mardookhpour [121] demonstrated that an optimal NS content, in the range of approximately 4–8%, is critical for limiting the degradation of mechanical properties of geopolymer concrete after exposure to 500 °C. In particular, the incorporation of 8% NS reduced the loss of the elastic modulus from about 42% to 33%, indicating improved stability of the material’s elastic skeleton under high-temperature conditions [121]. At the same time, a significant increase in ultrasonic pulse velocity (UPV) was observed, reaching up to ~40% in NS-modified specimens. This increase reflects a reduced density of microcracks as well as enhanced homogeneity and densification of the microstructure, confirming the effective role of NS in densifying the geopolymer matrix and stabilizing the ITZ after thermal exposure [121].

Other research increased this temperature and showed that NS enhances thermal resistance up to approximately 700 °C; above this temperature, a decrease in strength and microcracking is observed [122]. However, at 800–1000 °C gel dehydration and dehydroxylation lead to a reduction in load-bearing capacity, although mixes with an optimal nano-additive content still perform better than reference specimens [122]. This mechanism was also confirmed in other research, pointing to the limitation of material structure degradation at high temperatures [123]. In turn, Zhang et al. [124] reveal some mechanisms behind NS behavior in elevated temperatures up to 900 °C [124]. In fly ash–GGBFS-based geopolymer matrices reinforced with carbon fibers, NS remains highly active and thermally stable up to approximately 600–800 °C due to its high melting temperature. Within this range, NS enhances matrix densification, improves fiber–matrix bonding, and partially compensates for the progressive degradation of carbon fibers by strengthening the surrounding geopolymer gel [124]. At temperatures exceeding 800 °C, where carbon fibers undergo oxidation and lose their load-bearing function, NS plays a critical protective role by filling pores generated by fiber decomposition and by promoting partial microstructural sealing. This results in refined air-void characteristics, including shorter chord lengths and a reduced fraction of macropores, compared to matrices without NS [124]. Consequently, the synergistic combination of carbon fibers and NS provides superior high-temperature performance compared to either additive used alone, particularly in the 800–900 °C range [124].

Vanitha et al. [22] also show the benefits from the incorporation of NS beyond this temperature [22]. The incorporation of NS enhances thermal stability, as evidenced by lower mass loss in TGA, reduced thermal shrinkage (≈−1.1% compared to −1.4% for the reference), and retention of mechanical load-bearing capacity even after exposure to 800–1000 °C [22]. These improvements are associated with high-temperature phase transformations leading to the formation of refractory silica phases such as cristobalite and tridymite, which densify and further reinforce the geopolymer matrix, thereby improving its structural integrity under extreme thermal conditions [22]. In turn, Guo et al. [67] demonstrate that the NS-based sol–gel cementitious material retains high mechanical integrity even after exposure to extreme temperatures up to 1200 °C, with compressive strength remaining above 50 MPa, whereas ordinary Portland cement loses almost all load-bearing capacity [67].

The other works also confirm that NS also allows for improved mechanical behavior of geopolymer systems at elevated temperatures by promoting gel densification and viscous sintering, which counteract thermally induced cracking and porosity growth, leading to compressive strength values as high as 139 MPa at 1000 °C and a strength retention of up to 264% relative to ambient conditions [42]. As temperature increases, NS-rich matrices exhibit superior stiffness and strength retention due to the formation of thermally stable, highly cross-linked aluminosilicate and ceramic-like phases, accompanied by a reduction in total porosity to approximately 16% after high-temperature exposure [42]. However, NS does not significantly enhance the deformability of the material, but primarily contributes to an increase in load-bearing capacity [125]. At elevated temperatures, the beneficial effects of NS are offset by increased pore pressure and unfavorable phase transformations. As a result, the performance of NS-containing mortars at high temperatures reflects a balance between microstructural densification induced by NS and the material’s ability to accommodate thermal stresses through relaxation mechanisms [126].

Despite the presented research, there are still some promising directions in high-temperature studies. The behavior of nano-modified geopolymer concrete under high temperatures and fire exposure has not been sufficiently investigated, particularly with respect to spalling mechanisms [4].

### 8.3. Influence of NS on the Mechanical Properties in Hybrid Systems

Some provided works also confirm that NS can effectively work to improve mechanical properties in multi-component composites. Murugesan et al. [79] investigated a hybrid nano-engineered geopolymer composite incorporating 5% NS together with graphene oxide and CNTs, focusing on compressive and flexural mechanical performance under accelerated microwave curing [79]. The results showed that the addition of NS led to a compressive strength of about 120 MPa at 24 h and ~160 MPa at 28 days, corresponding to an increase of approximately 25–30% compared to comparable geopolymer systems without NS. In addition, flexural strength increased from ~6 MPa to ~12 MPa (≈100% improvement), which was attributed to microstructural densification, enhanced geopolymer gel formation, and reduced crack propagation enabled by NS [79].

Wu et al. [102] show that NS is a key enabler for fully exploiting the reinforcing potential of fiber systems, as its presence stabilizes the material response throughout the entire range of low-cycle fatigue loading. Acting at both the micro- and nano-structural levels, NS significantly enhances matrix densification and fiber–matrix interfacial bonding, thereby delaying microcrack initiation and slowing crack propagation. As a result, geopolymer composites incorporating 2% steel fibers and 0.2% polypropylene fibers exhibited over a 600-fold increase in fatigue life compared with plain geopolymer concrete at a stress level of 0.7, with NS contributing by refining the matrix microstructure, delaying microcrack initiation, and ensuring more effective stress transfer to the fibers throughout cyclic loading [102]. This synergy enables the design of high-strength, fatigue-resistant, and low-carbon geopolymer composites, making them particularly suitable for structural applications subjected to repeated or cyclic loading [102].

Hu et al. [77] confirmed that NS significantly enhances both early-age and long-term compressive strength, acting more directly on strength development than steel fibers, which mainly contribute to toughness and crack control. This improvement is attributed to the nano-filler effect, acceleration of geopolymerization reactions, and intensified formation of C-S-H, C-A-H, and C-A-S-H gels, leading to a denser and more cohesive geopolymer matrix [77]. Compared with steel fibers, NS acts more directly on compressive strength development, as it modifies the geopolymer matrix itself by densifying the pore structure and enhancing gel formation, whereas steel fibers mainly improve strength indirectly by bridging cracks, redistributing stresses, and increasing toughness rather than significantly increasing matrix stiffness. As a result, steel fibers contribute primarily to post-cracking behavior and durability, while NS governs the intrinsic load-bearing capacity of the geopolymer composite [77].

The content of NS can also help with the monitoring of mechanical properties. Wang et al. [127] focused on self-sensing geopolymer composites designed as low-carbon, multifunctional materials for structural health monitoring, aiming to combine high mechanical performance with high strain-sensing sensitivity. A key objective was to clarify how the material composition and multi-scale conductive system affect electrical response under mechanical loading [127]. NS was shown to be crucial for refining the nanopore structure, enhancing ionic conductivity, and stabilizing the sensing signal, thereby enabling reliable and highly sensitive self-sensing behavior [127].

## 9. Impact on Water Interaction Properties, Durability and Functional Performance

### 9.1. Water Interaction Properties and Durability

In NS-modified geopolymer mortars, NS reduces water absorption and sorptivity by refining the pore structure, filling capillary voids, and forming a more compact, less permeable geopolymer gel network [73]. For example, Harika et al. [63] show that the incorporation of NS significantly reduces sorptivity by refining pore structure and densifying the geopolymer matrix, thereby limiting water and ion ingress. As a result, NS-modified geopolymer concrete exhibits enhanced durability, particularly improved resistance to sulfate attack and long-term degradation under aggressive environmental exposure [63]. Also, NS-modified fly ash–GGBFS geopolymers demonstrated high waterproofing performance, achieving water penetration resistance classes between W10 and W14, compared with lower resistance in unmodified specimens [128]. Exposure tests in 2% NaCl, 2% Na_2_SO_4_, seawater, and 2% H_2_SO_4_ environments showed that NS-containing samples experienced only a 10–15% reduction in compressive strength after three months, confirming good resistance to chloride- and sulfate-rich media. Furthermore, the densified matrix and refined ITZ produced by NS were reported to effectively limit aggressive ion ingress, thereby contributing to improved protection of steel reinforcement against corrosion in aqueous chloride and sulfate environments [128].

The incorporation of NS significantly reduced water sorption and capillary transport in geopolymer concrete, as evidenced by an approximately 50% decrease in sorptivity and markedly lower water absorption at the optimum dosage of 3 wt.% NS [62]. This pore refinement and reduced connectivity translated into enhanced resistance to aggressive acidic environments, where NS-modified specimens exhibited only ~8% mass loss and substantially lower strength degradation compared to the reference mix without NS [62]. Overall, the improved durability is attributed to matrix densification and the formation of a more continuous geopolymer gel that effectively limits fluid ingress and chemical attack [62].

NS significantly reduces porosity and the transport of aggressive media, which directly translates into improved durability [117]. It also enhances the impact resistance of geopolymer concrete under coupled wet–thermal and chloride salt conditions. At an NS content of 1.5%, the impact toughness, the number of impacts to first cracking, and the number of impacts to failure increased by more than two times compared with the reference geopolymer concrete without NS [129].

Khan et al. [64] confirm the increasing durability. The addition of NS dioxide improves resistance to chemical attack and chloride penetration by refining the pore structure and densifying the geopolymer matrix, thereby limiting the ingress of aggressive ions. This enhanced durability is attributed to the nano-filler effect and accelerated gel formation, which reduces connectivity of capillary pores and microcracks [64]. Also, Sharma et al. [130] confirmed that the incorporation of NS enhanced the durability and resistance to fluid and ion transport in geopolymer concrete by refining the pore structure and densifying the matrix. At the optimum dosage of 2% NS, sorptivity and water absorption were reduced, the chloride diffusion coefficient decreased by approximately 15%, and the RCPT charge dropped by about 27% after 90 days compared to GPC without NS [130]. Moreover, the 2% NS mix exhibited the best resistance to sulfate and acid attack, confirming that controlled NS addition effectively limits ingress of aggressive media and improves long-term durability [130].

In the RCPT, the charge passed for mixtures containing NS was reduced to approximately 970 coulombs, compared to about 1070 coulombs for the reference mixture, corresponding to a ~29% reduction in chloride ion penetration [114]. Water absorption decreased systematically with increasing NS content, and for 4–6% nano-SiO_2_ the reduction amounted to approximately 0.5–0.8 percentage points compared to the geopolymer concrete without the nano-additive [114]. In sulfate resistance tests, the greatest improvement was also observed at 4% NS, where the mass loss of specimens was about 6% and clearly lower than that of the reference mixtures. Chiranjeevi et al. [114] attribute these effects to the ability of NS to fill capillary pores and to promote the formation of additional binding products, mainly C-A-S-H gel, which results in a reduction in effective porosity and limits the transport of aggressive media within the geopolymer structure [114]. Other research confirms that NS markedly reduced water absorption to below 3%, significantly decreased the depth of water penetration, and lowered the Rapid Chloride Permeability Test (RCPT) charge to approximately 2800–4100 C [131]. This reduction in RCPT values indicates a substantial limitation of chloride ion transport through the geopolymer matrix, reflecting enhanced pore refinement and improved durability against aggressive environments [131].

The above properties are strongly influenced by the dosage of NS. Hombali and Selvam [115] notice that material durability increases systematically with NS content, with the lowest water sorptivity and chloride permeability observed at 3.0–3.5 wt.% NS, even though these mixes do not exhibit the highest mechanical strengths [115]. In contrast, peak mechanical performance is achieved at lower NS levels (around 2.5 wt.%), indicating a trade-off between strength optimization and long-term durability governed by NS dosage [115]. In turn, Chen et al. [93] provided a study on coal gangue–GGBFS-based geopolymer concrete modified with NS at dosages ranging from 0 to 2.5 wt% [93]. The results showed that NS significantly densified the microstructure and reduced porosity, effectively limiting the ingress of sulfate ions (SO_4_^2−^) into the material and delaying chemical degradation during sulfate exposure. The lowest rate of strength loss and the highest sulfate resistance were observed for specimens containing approximately 1.5% NS, indicating this content as optimal for improving the durability [93].

NS not only increases the durability of the standard geopolymer matrix, but also the hybrid nano-engineered geopolymer composite—for example, in combination with graphene oxide and CNTs [79]. For these composites, the results showed a reduction in water absorption to about 2.5% and porosity to ~12%, as well as mass loss below 1% after exposure to acidic (pH 3) and alkaline (pH 11) environments, indicating improved resistance to environmental degradation [79]. In addition, the composite retained approximately 75% of its compressive strength at 900 °C and exhibited enhanced impact durability, which was attributed to microstructural densification and stable gel formation promoted by NS [79]. In this case, the role of NS is mainly connected with microstructure modification, which enhances matrix densification and promotes the formation of a stable and continuous geopolymer gel network. In hybrid systems, NS additionally acts synergistically with graphene oxide and carbon nanotubes by improving their dispersion, strengthening interfacial bonding, and facilitating more efficient load transfer. As a result, the combined nano-reinforcement leads to reduced porosity, improved resistance to aggressive environments, and enhanced high-temperature stability.

Dhasarathan and Kumar [132] proved that NS significantly reduced the permeability of geopolymer ferrocement mortar, as evidenced by a marked decrease in water penetration depth, indicating a denser and less connected pore structure [132]. In addition, enhanced acid resistance was confirmed through immersion in hydrochloric acid (HCl) at pH = 2 for 28 days, where NS-modified specimens exhibited lower mass loss and smaller reductions in compressive strength compared with the reference mix [132]. These improvements are attributed to pore blocking and matrix densification induced by NS, which limit fluid ingress and slow chemically induced degradation.

Interesting aspects are also introduced in the investigation presented by Ababneh et al. [133]. They show improved steel–geopolymer bonding after corrosion in kaolin-based geopolymer concrete reinforced with conventional ribbed steel rebars (Ø14 mm) when the bars were protected by an NS coating applied via dip-coating. Pull-out tests showed that specimens with NS-coated reinforcement achieved about 82% higher stiffness and 75% higher load-carrying capacity and ductility, along with a ~45% lower damage index (DI) compared to specimens with uncoated corroded steel [133]. These results confirm that NS coatings significantly enhance interfacial durability and mechanical performance of steel-reinforced geopolymer concrete under corrosive conditions [133].

Based on the study by Liu et al. [134], NS delays the negative effects of carbonation and stabilizes the microstructural development of geopolymer concrete over time. In accelerated carbonation tests (up to 56 days, 5% CO_2_), concrete containing 1.8 wt.% NS consistently showed a lower carbonation depth than the reference mix, with a reduced carbonation rate (slope ≈ 0.071 mm/day vs. 0.081 mm/day without NS) [134]. At the same time, NS shifted the inflection point of strength and stiffness development from 7 days to 14 days, indicating prolonged and more stable geopolymerization during carbonation [134]. This stabilization was associated with a denser pore structure, reflected in a porosity reduction from ~20.7% to ~17.5% and a lower average pore size (from ~34 nm to ~29 nm), which limited CO_2_ ingress and slowed microstructural degradation [134].

NS-modified geopolymers exhibited enhanced durability under aggressive environmental conditions, including sulfate attack and freeze–thaw cycling [120]. Compared with the reference material, the incorporation of 2% NS reduced water absorption by about 11% and resulted in smaller mass loss and strength degradation after coupled sulfate and freeze–thaw cycles [120]. This improved durability is attributed to lower porosity, restricted penetration of SO_4_^2−^ ions, and a more stable and compact gel structure [120]. Hu et al. [77] investigated freeze–thaw performance, subjecting the geopolymer composites to 275 freeze–thaw cycles ranging from −45 °C to +22 °C, after which compressive strength, flexural strength, mass loss, and microstructural damage were assessed [77]. The results showed that specimens containing approximately 1.0 wt% NS, especially in combination with steel fibers, exhibited minimal strength degradation and very low mass loss (below 1%), even after severe cycling [77]. It was concluded that NS significantly enhances freeze–thaw resistance by densifying the pore structure, reducing water migration, and mitigating microcrack development, thereby improving the long-term durability of geopolymer composites in cold environments [77].

Also, Deng et al. [135] confirm that NS enhances freeze–thaw resistance: after nine freeze–thaw cycles, samples with 3 wt% NS retained load-bearing capacity and showed much lower compressive strength loss than NS-free geopolymer-stabilized soils [135]. At the same time, frost heave and thaw settlement were strongly suppressed, with total vertical deformation reduced by more than 60% at 3 wt% NS. These improvements are attributed to pore filling and microstructural densification by NS, accelerated geopolymerization and gel formation, and a shift from free to bound water, which limits ice lens growth, reduces frost-induced cracking, and mitigates damage accumulation during repeated freeze–thaw cycles [135]. The other research also confirms that NS enhances thermal resistance and mechanical stability after repeated heating–cooling cycles [136].

Some other sorption aspects were investigated by Liang et al. [119]. They researched how NS modifies the adsorption of Cu^2+^ ions in porous geopolymer adsorbents made from fly ash and slag [119]. The study shows that although NS initially reduces Cu^2+^ adsorption due to pore filling and reduced porosity, higher NS contents promote the formation of N–A–S–H gels, which significantly enhance Cu^2+^ adsorption despite a denser matrix [119]. Experimental results supported by molecular dynamics and Density Functional Theory analyses demonstrate that N–A–S–H phases exhibit stronger physical interactions and lower Cu^2+^ mobility than C–A–S–H, confirming that NS improves Cu^2+^ uptake mainly by altering gel chemistry rather than by increasing surface area [119].

### 9.2. Other Functional Properties

Thanks to NS additives, geopolymers can gain a number of useful functional properties. One of them is self-healing potential. The study provided by Yuan et al. [137] investigated self-healing of geopolymer composites reinforced with PVA fibers, focusing on cracks of about 70–80 µm, which cannot be fully healed by conventional autogenous mechanisms [137]. The specimens were healed under water/air, air, and NS solution/air cycles, as well as with 0.5% NS added to the matrix, and the results showed that external application of NS solution was the most effective, leading to complete crack closure and recovery of up to 91.6% of fiber-bridging stress. The healing was attributed to NS acting as nucleation sites for C-(N)-A-S-H gel formation, significantly improving crack sealing and fiber/matrix interface recovery compared to internal NS addition alone [137]. This investigation shows that NS is significantly more effective as an externally applied crack-healing agent than as a conventional matrix additive, because its availability at later stages governs the intensity of healing reactions.

In application potential, the prevention of efflorescence, which is a common problem in geopolymers, can be increased. NS refines the pore structure of geopolymer concrete, leading to reduced sorptivity and permeability. As a result, the transport of free alkalis toward the surface is effectively limited, which significantly mitigates alkali migration and the formation of surface efflorescence. NS is identified as an effective additive for mitigating efflorescence in the investigated geopolymer system [138]. In particular, the investigation provided by Zhang et al. [138] shows that NS strongly refines the pore network, reducing the dominant pore size from approximately 26 nm to below 10 nm, which significantly restricts the migration of pore solution and alkaline ions [138]. This blocking of interconnected transport pathways, rather than a simple reduction in total porosity, is the key mechanism responsible for the suppression of efflorescence [138]. The mechanisms behind this phenomenon were also investigated by Guan et al. [18]. Ion mobility and structural stability were assessed using mean square displacement analysis within reactive molecular dynamics simulations to quantify the diffusion of Na^+^ ions and water molecules [18]. The results showed that Na^+^ and H_2_O diffusion is lowest in the ITZ and consistently lower in NS-modified NASH than in neat NASH, with the Na^+^ diffusion coefficient reduced to about 58% at Si/Al = 1.5. This enhanced immobilization of alkali ions implies reduced leaching and efflorescence risk, leading to improved chemical durability of the geopolymer [18].

In the case of immobilization, research in this area was also conducted by Ma et al. [139]. They show that NS significantly enhances the immobilization of heavy metals in geopolymer matrices by reducing the leaching of Cd, Cr, Cu, and Pb and increasing the proportion of these metals in chemically stable forms [139]. The presence of NS improves the effectiveness of solidification and stabilization during early curing stages. This behavior is mainly attributed to matrix densification and reduced permeability, which limit metal transport. In addition, heavy metal ions are incorporated into C–S–H and C–A–S–H gels and further immobilized through adsorption and ionic substitution within the geopolymer network [139]. In turn, Dassekpo et al. [140] designed a solution for radioactive contamination remediation in marine environments by embedding NS into a porous geopolymer concrete matrix [140]. NS derived from waste glass acts as the fundamental building block of a hydrothermally synthesized analcime zeolite (NaAlSi_2_O_6_·H_2_O), providing a mesoporous structure (~15–22 nm) and high specific surface area that enable efficient ion exchange and chemisorption of Sr^2+^ and Cs^+^ [140]. Importantly, the chemically stable Si–O–Al/Si–O–Si framework remains structurally intact after adsorption, meaning that NS functions as an active immobilization phase, not merely as a microstructural densifier, ensuring long-term radionuclide fixation under marine conditions [140].

Another connected property is electrical conductivity. With increasing NS content, the electrical resistivity of the geopolymer concrete increased while the bulk electrical conductivity decreased, indicating a denser matrix and hindered ionic transport [76]. This effect was most pronounced at 3–4% NS, suggesting enhanced microstructural refinement at these dosages [76]. However, in more complex systems, the incorporation of NS can give opposite results; in carbon fiber-based geopolymer composites it leads to a reduction in electrical resistivity of up to ~60%, demonstrating its decisive role in promoting a more homogeneous and better-connected conductive network of carbon fibers within the matrix [109]. This effect arises from the NS-induced improvement in fiber dispersion and interfacial interactions, which facilitate the formation of continuous electrical pathways and minimize local discontinuities. In practical terms, the NS-driven enhancement of electrical conductivity improves the robustness and sensitivity of multifunctional composites, making them particularly suitable for self-sensing, damage monitoring, and electrically functional construction materials [109]. In turn, Janowska-Renkas et al. [141] showed that hydrophobic NS reduces the electrical resistivity of geopolymer composites, particularly in systems with a high fly ash content, indicating improved electrical conductivity [141]. In contrast, hydrophilic NS tends to increase resistivity, leading to a deterioration in conductive performance. The authors therefore conclude that the type of NS is a key factor governing the electrical conduction behavior of geopolymers [141].

Another interesting observation is the impact of NS on formed geopolymers. NS contributes to foam stabilization by refining the pore structure and reducing the risk of foam collapse during geopolymer setting [142]. Consequently, it is identified as one of the most promising modifiers for foam geopolymers, although further research is required to optimize dosage, ensure effective dispersion, and enable industrial-scale application [142].

In turn, Figiela et al. [143] demonstrated that NS in geopolymer composites does not act as a direct antibacterial agent; instead, by modifying the microstructure, alkalinity, and surface conditions, it indirectly limits microbial growth, particularly through synergistic interactions with other additives, making it a valuable component for materials intended for water-related infrastructure applications [143].

### 9.3. Overview of Influence of NS on Geopolymer Performance

Table 6 synthesizes the key findings from the literature reviewed in the preceding sections regarding the influence of NS on selected properties of geopolymer composites. It provides a concise comparative overview of reported physical, chemical, and interfacial effects of NS addition, highlighting the main trends and mechanisms identified across studies.

It should be noted that the effects of NS in geopolymer systems are highly multifactorial, depending on precursor chemistry, activator composition, NS dosage, dispersion quality, and curing conditions. Consequently, while many studies report performance enhancement, deviations or even adverse effects are observed in certain geopolymer formulations, underscoring the system-specific nature of NS modification.

## 10. Application-Oriented Case Studies

By enhancing key performance attributes through controlled interfacial and matrix refinement, NS-modified geopolymers open new application pathways beyond conventional structural uses, addressing functional, durability-driven, and advanced construction demands. Table 7 illustrates how NS modification enables the translation of tailored geopolymer microstructures into a broad and expanding range of practical applications.

Among the different applications, a very frequently raised issue is environmental influence. Geopolymers are traditionally considered sustainable materials because of the reduced energy consumption, carbon footprint, and possibility of using by-products for manufacturing [151,152]. For example, Liang et al. [153] also demonstrate the eco-efficiency of NS additive to that waste geopolymer powder derived from demolished alkali-activated geopolymer materials, such as GGBS-based, GGBS–fly ash-based, and fly ash-based geopolymers [153]. This material is not sufficiently reactive to replace cement without deteriorating mortar properties. However, the incorporation of NS as an activating additive effectively enhances the microstructure, mechanical strength, and durability of waste geopolymer powder-blended mortars, making this approach a viable and sustainable materials strategy [153].

In turn, the life-cycle assessment (LCA) provided by Deng et al. [135] shows the negative impact of NS [135]. Although the addition of NS increases both the cost and carbon footprint, the article shows that this drawback is offset by a much larger gain in mechanical performance and durability, especially under freeze–thaw cycles [135]. Specifically, the authors carried out quantitative sustainability analyses, including a carbon emission index and an economic efficiency index, which demonstrated that 3 wt% NS provides the lowest environmental and economic indices after multiple freeze–thaw cycles, confirming that the performance benefits outweigh the added environmental and financial burdens [135].

Other authors, who also used an LCA, demonstrated that engineered geopolymer composites based on fly ash and ground granulated blast furnace slag exhibit a substantially lower carbon footprint than conventional OPC concrete. The inclusion of NS at low, optimized dosages does not offset these environmental benefits, as its contribution to embodied emissions remains marginal relative to the reductions achieved by eliminating Portland cement [60]. Overall, sustainability is primarily governed by the use of low-cement or cement-free binders, locally sourced supplementary materials, and careful optimization of mix composition [60]. The divergent results indicate the need for further research into LCAs and large-scale industrial implementation [66].

## 11. Challenges and Limitations

**Figure 8:**
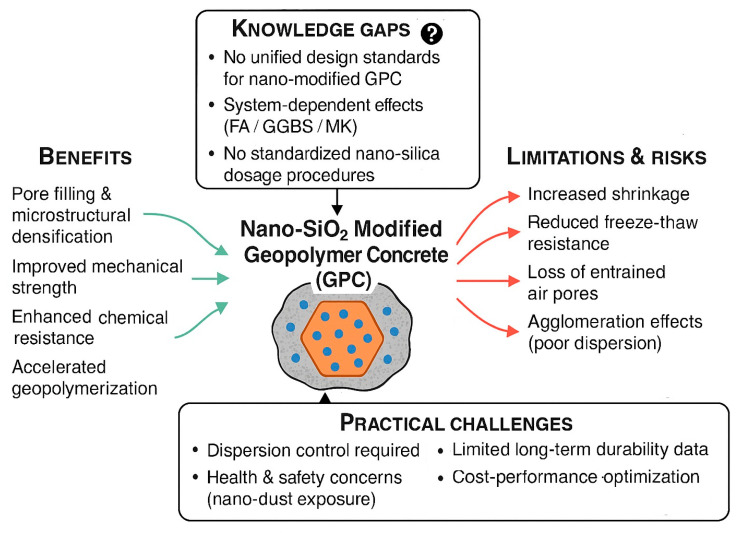
Schematic overview of the beneficial effects, limitations, and unresolved challenges associated with NS incorporation in geopolymer concrete systems.

The performance of NS is highly system-dependent and influenced by precursor chemistry, dispersion quality, curing conditions, and environmental exposure. Figure 8 presents the most important beneficial effects, limitations, and unresolved challenges associated with NS incorporation in geopolymer concrete systems.

Despite the large number of investigations into NS, there are still some knowledge gaps in this area. The main reason is that the final material behavior is influenced by a lot of factors [55]. There is no universal effect of NS in geopolymer systems, as its influence strongly depends on the type of precursor material (fly ash, GGBS, or metakaolin), chemical composition, curing conditions, and dosage. As highlighted by several reviewed studies, NS may accelerate geopolymerization and improve strength or durability in one system, while producing a different or even an opposite effect in another, which underscores the system-specific nature of its action [25]. Consequently, there is no unified or standardized procedure for determining the optimal dosage of NS in geopolymer systems [25]. The absence of dedicated design codes and standardized guidelines for geopolymer concrete incorporating nanomaterials limits confidence in structural design and hinders large-scale practical implementation [4].

Some limitations in current research are connected with the effects of nanomaterials on drying, shrinkage and resistance to freeze–thaw cycles, which remain inadequately understood, as matrix densification may increase shrinkage sensitivity and reduce entrained air content required for frost resistance [4]. Kumar et al. [4] pointed out that a potential risk associated with the use of NS is increased shrinkage and reduced resistance to freeze–thaw cycles, mainly due to matrix densification and the reduction in entrained air pores needed to accommodate ice formation [4]. Another risk in practical implementation is connected with the fact that the effectiveness of NS is strongly influenced by its degree of dispersion, since inadequate dispersion or agglomeration may diminish its beneficial effects on the material properties [154].

Key practical challenges associated with NS application remain unresolved, particularly issues related to particle dispersion, material cost, and health and safety concerns during handling and use of nanomaterials [25]. In the case of scale-up, potential application-controlled dispersion to prevent agglomeration is a crucial issue. Achieving uniform dispersion of nanomaterials is critical, as particle agglomeration can negate their beneficial effects; therefore, controlled dispersion techniques, such as sol–gel processing or pre-dispersion in liquid media, are often necessary [4]. The important issues are also health and safety concerns. Handling nanomaterials poses occupational health risks due to their fine particle size, dust generation, inhalation hazards, and potential long-term nano-toxicity, requiring strict safety protocols and personal protective equipment [4]. For large-scale applications and standardization of processes, the crucial problem is insufficient long-term durability data. Comprehensive long-term durability studies are still scarce, especially regarding aging mechanisms, chemical resistance over extended service periods, and performance under sustained environmental loading [4,112].

From an economic perspective, NS-modified geopolymer concrete was found to be cost-competitive with conventional OPC concrete, as the use of GGBS as an industrial by-product significantly offsets material costs [130]. Owing to its enhanced durability and reduced susceptibility to chemical attack and chloride ingress, NS-GPC is expected to require less repair and maintenance over its service life, providing clear life-cycle cost benefits. In addition, the replacement of cement with GGBS markedly reduces the carbon footprint, strengthening the economic and environmental viability of NS-enhanced GPC as a sustainable construction material [130].

## 12. Future Directions

NS is one of the most promising modifiers for foamed geopolymers, requiring further research into optimal dosage, dispersion, and industrial scalability [142]. Indwar et al. [1] claim that NS is currently considered the most promising nanomaterial additive for engineering-scale applications of geopolymer concrete. However, further research is required to address issues related to long-term durability, performance at elevated temperatures, and the scalability of the technology [1]. In turn, Shumuye et al. [5] claim that further research should focus on standardizing NS dosing and dispersion methods, as well as on assessing the long-term durability of geopolymers incorporating NS. In addition, greater attention should be given to applications in 3D printing and low-carbon structural systems, where NS may offer significant performance and sustainability benefits [5]. All of these directions point to rather short-term perspectives and emerging directions in the study area.

In the long-term development of these compositions, some other directions seem to be more promising. One of the most promising directions is the development of advanced composites with NS. An example is the research of Yu et al. [92]. They showed that NS can be a valuable additive in functional composites. In order to address the degradation of mechanical strength and thermal conductivity caused by unmodified PCM additions, NS is applied to the surface of CA-PA/RMHM to densify the interfacial transition zone and limit porosity growth, which are the main mechanisms responsible for performance loss in cement mortars containing bare PCM capsules [92]. The purpose of introducing an NS coating is to suppress these negative effects while simultaneously maintaining the latent heat storage capability of the PCM system. By targeting ITZ strengthening and pore refinement, NS enables a more favorable balance between thermal energy storage efficiency and acceptable mechanical performance in PCM-modified cementitious composites [92].

Another research area that requires more study is the synergy effect between different additives. Such a kind of effect is reported in some investigations. For example, Hu et al. [77] demonstrated a synergistic effect between NS and steel fibers, where NS densifies and strengthens the geopolymer matrix while steel fibers bridge cracks and redistribute stresses, resulting in superior mechanical performance and freeze–thaw resistance compared to the use of either modifier alone [77]. In turn, Raut et al. [66] analyzed the synergy between NS and other nano-additives. They investigated a hybrid nano-engineered geopolymer composite incorporating NS combined with graphene oxide (GO) and carbon nanotubes (CNTs), focusing on mechanical performance, durability, and multifunctionality [66]. Experimental results showed that NS–GO synergy improves interfacial bonding and crack-bridging efficiency, while the NS–CNT system enables a dense matrix and a continuous conductive network, supporting reliable self-sensing behavior [66]. Overall, this synergistic nano-engineering strategy produced a multifunctional geopolymer material with superior strength, enhanced durability, and intelligent monitoring capability, exceeding the performance of single-nano-admixture systems [66]. Wang et al. [155] showed the synergy in coating applications [155]. A stable nano-SiO_2_–TiO_2_ coating was formed on the geopolymer surface, exhibiting strong adhesion and no visible cracking [155]. The coated material showed high hydrophobicity, with a water contact angle of up to 118°, and pronounced photocatalytic activity, as confirmed by the degradation of organic dyes (methyl orange), oily contaminants (oleic acid), and effective removal of dust (red mud). NS plays a critical role by improving TiO_2_ dispersion, stabilizing the coating through siloxane bonding, enhancing hydrophobicity, and synergistically reducing the effective band gap of TiO_2_, thereby improving UV utilization and accelerating photocatalytic reactions; the optimal performance was achieved at approximately 5.8 wt% nano-SiO_2_–TiO_2_, enabling promising applications in building façades, architectural elements, and urban infrastructure with enhanced durability and low maintenance requirements [155].

Yang et al. [20] demonstrated that NS is a key functional additive enabling the combination of high radiative cooling efficiency with exceptional resistance to extreme temperatures [20]. As a result, phosphate-activated geopolymer–NS coatings represent a robust inorganic alternative to conventional organic cooling systems, particularly suitable for infrastructure and buildings exposed to intense solar radiation and high service temperatures [20]. From an optical perspective, NS enhances infrared emissivity through phonon–polaritonic resonance around 9.7 µm, while simultaneously optimizing pore-scale morphology to improve light scattering via the Mie effect. Importantly, NS also stabilizes the optical performance of the coating even after prolonged high-temperature exposure, ensuring long-term durability and functionality [20].

The modern area that also requires more in-depth research is AI studies. Sharma et al. [156] showed that combining materials science knowledge with interpretable AI frameworks enables not only highly accurate prediction of geopolymer concrete properties, but also transparent identification of how key variables—such as NS dosage—interact with curing and activator parameters [156]. By integrating explainable deep learning, uncertainty quantification, and multi-objective optimization, the AI approach provides actionable insight into the role of NS and supports rational, low-carbon mix design of high-performance geopolymer concretes [156].

Computer modeling can be especially challenging in the case of nano-additives. Khan et al. [64] showed that the incorporation of NS introduces strong non-linearity into the geopolymer concrete system, as the compressive strength increases moderately at 1% NS, reaches an optimum at 2%, and decreases at 3% due to nanoparticle agglomeration and poor dispersion [64]. Such threshold-dependent behavior is difficult for an Artificial Neural Network (ANN) to capture accurately, leading to smoothed predictions around the optimum. In contrast, the Adaptive Neuro-Fuzzy Inference System (ANFIS) effectively identifies effective NS ranges through fuzzy rules, resulting in lower prediction errors, as well as higher performance indices [64]. Also, ANN was used for modeling by Gupta and Yadav [65]. They applied the hybrid Finite Element Interpolated Neural Network (FEINN), which integrates Finite Element Method (FEM)-based fracture mechanics with neural-network interpolation, enabling physically informed learning of material behavior. Within this model, NS was treated as a critical input variable whose influence on mechanical strength and fracture behavior is learned and quantitatively captured from experimental data, resulting in improved prediction accuracy and more reliable assessment of crack initiation and propagation [65].

## 13. Conclusions

The current review shows that NS offers a pathway to tailored, low-carbon geopolymers with superior microstructure–performance relationships aligned with sustainable construction goals. It functions as an active nanoscale design tool in geopolymer systems, enabling deliberate control of geopolymerization kinetics, pore structure refinement, and ITZ densification, which collectively translate into enhanced mechanical performance and durability. However, its effectiveness is strongly system- and dosage-dependent, requiring precise optimization of precursor chemistry and dispersion to avoid agglomeration-induced drawbacks and to fully exploit its potential in advanced and sustainable geopolymer applications. In particular, based on the review, it is possible to formulate some main conclusions:

- Nano-silica fundamentally alters geopolymer systems by acting as an active nanoscale design parameter, enabling deliberate control of geopolymerization kinetics, gel chemistry, and microstructural evolution rather than serving as a passive filler.
- The primary contribution of NS occurs at the reaction and nucleation stage, where highly reactive amorphous SiO_2_ accelerates dissolution–polycondensation processes and promotes the formation of continuous, highly cross-linked N-A-S-H and C-(N)-A-S-H gels.
- At the microstructural level, NS consistently induces pore structure refinement, reduces connectivity of capillary pores, and shifts porosity toward gel-scale domains, which directly governs permeability, sorptivity, and durability.
- The presence of NS transforms the ITZ from a weak or passive region into a dense, chemically active, and load-bearing zone, fundamentally redefining ITZ behavior in geopolymer composites compared to OPC systems.
- Improvements in mechanical performance—including compressive, tensile and flexural strength; fracture toughness; fatigue resistance; and high-temperature stability—are shown to be direct consequences of nanoscale gel densification and ITZ engineering.
- NS has a critical enabling role in advanced geopolymer applications, including fiber-reinforced composites, 3D-printed materials, repair mortars, functional coatings, smart/self-sensing systems, self-healing concepts, and extreme-environment applications such as ISRU-based construction.
- The review confirms that NS performance is highly dosage- and system-dependent, controlled by precursor chemistry, calcium availability, the Si/Al ratio, particle size, dispersion quality, and the curing regime; consequently, no universal optimal dosage can be defined.
- Overall, nano-silica enables a microstructure-driven design paradigm for geopolymers, where nanoscale mechanisms are systematically translated into predictable macro-scale performance, supporting the development of durable, multifunctional, and low-carbon construction materials.

## Figures and Tables

**Table 1:** The types of NS used in the geopolymer matrix.

No.	Type of NS	Size (Typical)	Description	Advantages	Disadvantages	Source
1	Colloidal NS	10–50 nm (typically 10–30 nm in stable sols)	A dispersion of NS in water (stable sol) ensures good spreading of particles in the matrix and high chemical reactivity. It exhibits a strong nucleation effect and significantly accelerates early-age reactions.	Easy dosing, no dust formation, minimal agglomeration.	Higher cost than dry powder, limited long-term stability of the dispersion.	[30,31]
2	NS in dry powder form	5–100 nm (often strongly agglomerated)	Amorphous SiO_2_ nanoparticles (often produced via flame synthesis) that have a very high specific surface area. Requires intensive dispersion methods (mechanical mixing, ultrasonication).	High reactivity, ease of storage.	Strong tendency to agglomerate, dosing difficulties.	[32,33]
3	Surface-modified NS	Typically similar to base NS (~10–100 nm)	NS functionalized (e.g., with silane agents) provides improved compatibility with organic or inorganic matrices. Less frequently used in geopolymers, more common in polymers and hybrid composites.	Controlled interfacial interactions.	Higher cost, reduced chemical reactivity of silica.	[5,34,35]
4	In situ NS	20–60 nm (variable depending on synthesis conditions)	Generated directly within the system (e.g., from silicate precursors), this ensures a very homogeneous dispersion at the nanoscale.	No agglomeration, excellent integration with the gel network.	Complex process control.	[36,37,38]
5	Waste-derived/bio-based NS	10–100 nm (high variability depending on precursor and process)	Produced, for example, from rice husk ash or other silica-rich wastes. Typically amorphous, with variable purity and particle size.	Low cost, environmental benefits.	Variable quality, lower reproducibility of results.	[39,40,41]

**Table 2:** Types, properties, and dosage ranges of NS used in geopolymer systems.

No.	Type of NS	Average Particle Size [nm]	SiO_2_ Purity [%]	Typical Dosage Range	Dispersion	Main Effects of NS	Source
1	NS powder	75 nm	90.5%	3 wt.% of binder	Dry dispersion in the solid binder phase via mechanical mixing	Increase in mechanical properties; microstructural densification and particle interlocking; more homogeneous geopolymer matrix	[61]
2	NS powder	40 nm	95.8%	0–4 wt.% of binder (optimum: 3 wt.%)	Dry dispersion in the solid binder phase via mechanical mixing	Increase in mechanical properties; reduction in sorptivity and water absorption; improved acid resistance; denser matrix, fewer pores, improved geopolymer gel continuity	[62]
3	NS powder	17 nm	95%	2–3 wt.% of binder	Dry dispersion in the solid binder phase via mechanical mixing	Accelerated dissolution of Si and Al, increased formation of gel and stronger Si–Al bonding; pore filling and matrix densification; reduction in sorptivity and water absorption; improved sulfate resistance; more compact and homogeneous microstructure	[63]
4	NS powder	Ultrafine nanoscale particles (exact diameter not specified)	95–98%	1–3 wt.% of GGBS ^1^ (optimum: 2 wt.%)	Dry dispersion in the solid binder phase via mechanical mixing; dispersion assisted by alkaline activator solution and superplasticizer	Acceleration of geopolymerization reactions; increase in mechanical properties; reduction in setting time; densification of the geopolymer matrix; improved resistance to chemical attack and chloride penetration	[64]
5	NS powder	≈30 nm	99.7%	0–1.0 wt.% of binder (optimum: 1.0 wt.%)	Water–superplasticizer solution, followed by incorporation into the alkaline activator	Matrix densification; increased gel formation, increase in mechanical properties; enhanced crack resistance and fiber–matrix bonding; reduction in workability and higher water demand	[65]
6	Colloidal NS produced via sol–gel synthesis	10–30 nm	99%	0.16–0.32 wt.% of solid SiO_2_	Aqueous silica sol (water-based colloidal dispersion)	Enhanced geopolymerization kinetics; increased compressive strength; refined pore structure; reduced nanoscale heterogeneity; improved gel continuity	[56]
7	Colloidal NS	10–50 nm	>99%	5 wt.% of binder	Alkaline geopolymer precursor slurry, with dispersion achieved through ultrasonication in liquid media followed by mechanical milling to ensure homogeneous distribution	Acceleration of geopolymerization via nucleation effects; microstructural densification and porosity reduction; enhancement of early and long-term compressive and flexural strength; improved thermal stability, durability, and impact resistance, especially in synergy with GO and CNTs	[66]
8	In situ-grown NS	Approximately 53.96 nm	Not specified	0.5–3.0 wt.% of fly ash (optimum: 2.0 wt.%)	In situ chemical anchoring on fly ash surfaces via Si–O–Si bonds	Enhanced geopolymerization and gel formation; refined pore structure—reduction in harmful pores (>50 nm); improved compressive strength; reduced nanoparticle agglomeration; slight impact on workability compared to commercial NS; more homogeneous microstructure	[38]

^1^ GGBFS—ground granulated blast furnace slag.

**Table 3:** Mechanisms of NS action on geopolymer microstructure at different length scales.

No.	Role of NS	Dominant Mechanism	Key Characterization Techniques	Source
NANOSCALE				
1	Primary reactive silica source enhancing geopolymerization.	Rapid dissolution of highly reactive amorphous SiO_2_ and increased availability of silicate species, leading to intensified polycondensation and higher gel cross-linking (N-A-S-H/(C,N)-A-S-H.	FTIR (Si–O–T band shifts), XRD (amorphous hump evolution), TGA/DTG (gel dehydration behavior).	[42]
2	NS acts as a nano-filler and nucleation enhancer, assisting geopolymer gel development at early stages.	Enhanced geopolymerization potential through nanoscale particle–gel interactions. Filling of nano- and sub-micro voids not accessible to fly ash and GGBFS particles.	Material characterization data (particle size, chemical composition).	[61]
3	NS acts as nano-fillers occupying nano- and sub-micro voids unavailable to fly ash or metakaolin. Serves as nucleation sites for aluminosilicate gel formation.	Acceleration and enhancement of geopolymerization via increased reactive silica surface and nucleation density.	Material specification (particle size, chemistry). Indirect confirmation through strength and durability gains.	[62]
4	Reactive silica source and nucleation sites for geopolymer gel formation.	Rapid dissolution of highly reactive Si species combined with heterogeneous nucleation of C-(N)-A-S-H gel, leading to enhanced gel compactness and continuity.	SAXS with Guinier analysis (gel compactness and pore size), AFM (surface topography, modulus mapping, correlation length).	[56]
	Pore-filling of submicron defects and nanoporosity in ITZs; pozzolanic reaction; nucleation effect.	Chemical reaction and nucleation-controlled densification (accelerating geopolymer gel (N-A-S-H/C-(N)-A-S-H formation at interfaces).	Thermogravimetric analysis (TGA), X-ray diffraction (XRD), nanoindentation.	[37]
5	Nucleation and densification agent for binding gel.	Provision of abundant nucleation sites for gel precipitation, resulting in a denser and more homogeneous aluminosilicate network.	FTIR, SEM (gel morphology), nanoindentation (local stiffness of paste).	[42]
MICROSCALE				
6	Micro-/nano-filler improving pore structure.	Physical filling of micro- and mesopores and refinement of pore size distribution, reducing capillary porosity.	Mercury Intrusion Porosimetry (MIP), SEM.	[42]
7	Improvement in interfacial transition zone (ITZ) quality.	Chemical and microstructural densification of the ITZ through enhanced geopolymer gel formation adjacent to aggregates.	Nanoindentation (ITZ modulus and hardness), SEM.	[42]
8	Denser and more compact geopolymer matrix with fewer visible pores. Reduced capillary pore continuity and crack paths. Improved gel homogeneity.	Micro-filler packing and secondary gel formation (additional aluminosilicate gel produced due to NS reaction).	SEM (qualitative microstructural analysis), water absorption test, sorptivity test (ASTM C1585).	[62]
9	Microstructure densifier and interfacial modifier.	Uniform precipitation and growth of geopolymer gel around precursor particles, improved particle–gel bonding, and pore filling at the microstructural level.	SEM (microstructure morphology), EDS mapping (Si and Al distribution), AFM roughness analysis (Ra, Rq).	[56]
10	Reduction in ITZ width (up to ~10 μm). Significant increase in elastic modulus of ITZs (up to ~96%). Transformation of harmful large pores into finer pores, improving mechanical continuity.	Microstructural densification and stiffness homogenization.	SEM, backscattered electron imaging (BSE), image-based porosity analysis.	[37]
11	Promotion of viscous sintering at high temperatures.	Facilitation of viscous flow and sintering of the geopolymer matrix above ~800 °C, leading to pore closure and structural compaction.	SEM (sintered morphology), MIP (porosity reduction), XRD (onset of crystalline phases).	[42]
MESOSCALE				
12	Contribution to the formation of thermally stable ceramic-like phases.	Transformation of amorphous aluminosilicate gel into stable crystalline phases (e.g., leucite, kalsilite) under high-temperature exposure.	XRD (phase identification), TGA/DTG.	[42]
13	Enhancement of macroscopic strength and thermal stability.	Indirect macro-scale strengthening through combined gel densification, ITZ improvement, pore refinement, and sintering.	Compressive strength testing, shrinkage measurements correlated with micro/nano analyses.	[42]
14	Improvement in macroscopic mechanical performance.	Translation of nano- and microscale densification into higher load-bearing capacity, improved stress transfer, and reduced weak zones within the brick body.	Compressive, tensile, and flexural strength testing.	[61]
15	Improvement in macroscopic mechanical properties. Enhanced acid resistance.	Translation of nano- and microscale densification into improved load transfer, reduced transport of aggressive agents, and higher structural integrity.	Compressive, splitting tensile, and flexural strength tests, mass loss and residual strength after acid exposure.	[62]
16	Mechanical performance enhancer.	Translation of nanoscale gel densification and microscale structural homogeneity into improved load transfer and mechanical integrity.	Compressive strength testing (early and later ages).	[56]
17	Improvement in mechanical properties. Enhanced water absorption and crushing index tests.	Improved interfacial bonding and stress redistribution.	Water absorption and crushing index tests. Compressive and flexural strength tests.	[37]

**Table 4:** Comparison of ITZ characteristics in OPC and geopolymer systems without and with NS.

Feature	OPC	Geopolymer	Geopolymer with NS
ITZ porosity	high	low–moderate	low
ITZ thickness	20–50 µm	thin/weakly defined (≈10–30 µm)	very thin/often indistinct (<10 µm) ^1^
ITZ character	passive	reactive	highly reactive
ITZ strength	weakest zone	comparable to the matrix	often higher than the matrix
Role of NS	none (CH, ettringite)	silica from the activator and precursor, partly influence ITZ	active ITZ designer (reactive Si + filler effect)

^1^ The literature more often refers to the “absence of a classical ITZ” rather than to a single numerical value.

**Table 5:** Comparison of mechanical strength improvements in NS-modified geopolymers.

No.	Precursor	NS Dosage	Compressive Strength	Tensile/Flexural Strength	Reported Effect	Source
1	Fly ash + GGBFS	3 wt.%	40% increase	Tensile +27%, flexural +33%	Significant improvement due to matrix densification	[62]
2	Geopolymer bricks	3 wt.%	41.5 → 45 MPa	Tensile: 3.35 → 4.5 MPa; flexural: 6.2 → 6.5 MPa	Improved particle interlocking and densification	[61]
3	Fly ash + GGBFS + SBA	4 wt.%	41–43 MPa	3.9 MPa tensile	+25–30% strength increase	[114]
4	SBA-based geopolymer (fly ash + GGBFS + SBA)	2.5 wt.%	+32%	+20–25% (tensile/flexural)	Improved ITZ and geopolymerization	[115]
5	Sludge-based geopolymer	2 wt.%	+108.2%	Flexural +69.9%	Highest improvement among nano-additives	[120]
6	Porous geopolymer adsorbent	2 wt.%	+63.5%	N/A	Improved structural stability	[119]

**Table 6:** Influence of NS’s selected properties on geopolymer composites.

No.	Aspect/Property	Effect of NS Addition	Dominant Mechanism	Key Benefits	Limitations/Risks
1	Fresh-state properties (workability, rheology)	Decreasing workability at higher dosages; increasing stability and yield stress at lower dosages	High specific surface area; increased water demand; particle packing	Improved shape stability; at low doses, possible improvement in grain packing; in some systems, increased spreading	Reduced flowability, strong dependence on dispersion; increased need for water
2	Rheological behavior (printability)	Enhancing yield stress and shape stability	Particle packing, thixotropy enhancement	Better printability	High dosage of NS can limit rheological properties
3	Setting and hardening	Acceleration or, in some systems, delay	Nucleation effect, enhanced dissolution and gel formation	Faster early strength development	Possible disturbance of reaction kinetics
4	Dimensional stability/shrinkage	Reduction in shrinkage	Structural densification	Improved dimensional stability, reduction in cracks after the curing process	At high doses, an increase in autogenic contraction is possible
5	Interlayer adhesion (3D printing)	Improved bonding between layers	Enhanced fresh-state cohesion	Good interlayer adhesion in 3D printed products	High dosage can cause agglomerations
6	Microstructure	Reduced porosity; increased homogeneity	Nano-filler effect, controlled gel growth	Denser and more uniform matrix; reduction in open porosity; improved bonding of gel phases (N-A-S-H/C-(A)-S-H)	Agglomeration may create weak zones; a negative effect due to improper dispersion
7	Compressive strength	Significant improvement; improvement in early-age strength	Matrix densification, pore refinement	Higher ultimate and early strength	Strength decreases beyond optimal dosage
8	Flexural/tensile strength	Moderate-to-great improvement	In composites, improved ITZ, stress transfer	Increased toughness and fracture resistance	Excess NS may reduce ductility
9	Fiber-reinforced systems (and other composites)	Improved crack bridging and interlayer adhesion	Enhanced cohesion and interface quality	Beneficial for strain-hardening composites; enhanced toughness and fracture toughness	Higher sensitivity to processing errors
10	Shrinkage and cracking	Reduced crack width	Stress redistribution, denser matrix	Improved dimensional stability	Risk of increased autogenous shrinkage at high dosages
11	Self-healing capacity	Enhanced crack sealing	Promotion of gel re-precipitation	Improved durability and service life	Effectiveness depends on microstructure quality
12	Durability (permeability, chemical resistance)	Improved resistance to water, chlorides and aggressive media	Reduced connected porosity	Lower water absorption; lower permeability; better chemical stability	Poor dispersion may increase sorptivity
13	Technological aspects	High efficiency at low dosages	High intrinsic reactivity	Material-efficient modification	Difficult and costly dispersion techniques
14	Environmental aspects	Indirect reduction in binder demand	Strength and durability enhancement	Potential reduction in structural carbon footprint	NS production is energy-intensive; a lack of complete LCA data for nano-systems
15	Reproducibility of results	Mechanisms well understood	—	Clear trends at optimal dosages	Large scatter in the literature results; no definitive “universal” dose

**Table 7:** Application-driven performance improvements enabled by NS-modified geopolymers.

No.	Application Area	Performance Requirements	Effect of NS	Key Benefits	Practical Limitations	Source
1	Reinforced geopolymer concrete slabs for structural elements, such as floor and roof slabs in buildings and infrastructure.	Adequate flexural strength, crack resistance, stiffness, ductility, and energy absorption capacity, together with reliable structural behavior.	NS significantly densifies the geopolymer matrix, reduces voids, delays crack initiation, and enhances load-carrying capacity, stiffness, and energy absorption under flexural loading.	Increased cracking load and ultimate flexural capacity; improvement in stiffness and energy absorption; better crack control and resistance to micro-strain development; improved overall structural efficiency and durability.	Potential issues related to dispersion, cost, and workability at higher NS contents; structural design guidelines for geopolymers with nano-additives are still not standardized.	[144]
2	Self-compacting geopolymer concrete paver blocks, designed for medium-traffic applications, such as pedestrian walkways, residential driveways, parking areas, and urban pavements.	High compressive, tensile, and flexural strength; low water absorption; good durability under ambient curing conditions; and sufficient structural integrity to resist cracking, abrasion, and environmental exposure during service.	NS enhances matrix densification and geopolymerization, leading to increases in mechanical properties; it simultaneously reduces water absorption and porosity; higher NS contents promote better particle packing and improved formation of binding gel phases.	Improvement in mechanical strength, reduced water absorption, improved durability, and the ability to produce high-performance, low-carbon paver blocks using industrial by-products (system is environmentally sustainable).	Excessive NS content increases water demand and cost, requires careful control of dispersion and mix rheology, and could potentially lead to workability issues if not balanced properly with superplasticizers.	[145]
3	Geopolymer repair mortars for rehabilitation and strengthening of reinforced concrete structures exposed to sulfate-rich aggressive environments, such as bridge piers, foundations, marine and underground infrastructure.	High bond strength to existing concrete substrate, adequate compressive strength, controlled shrinkage, and durability under sulfate attack, without the need for heat curing.	NS improves geopolymer performance by densifying the matrix, refining pore structure, and enhancing ITZ properties; increases shear and tensile bond strength.	Enhanced bond strength between repair mortar and concrete substrate; improved compressive strength and matrix uniformity; increased resistance to sulfate curing environments; denser and more durable microstructure; synergistic performance when combined with fiber content.	Increased drying shrinkage; sensitivity to NS dosage and dispersion method; requirement for fiber optimization to mitigate shrinkage; long-term durability still requires further validation.	[146]
4	Flame-retardant and UV-resistant protective coatings for wood-based construction materials (e.g., plywood) used in buildings and transportation.	High flame retardancy, thermal stability at elevated temperatures, resistance to UV-induced aging, and durability of protective performance after long-term exposure.	NS densifies the geopolymer coating, fills pores and microcracks, enhances char formation, promotes stable Si–C–P residues, increases activation energy of thermal decomposition, and improves UV shielding through reflection and absorption.	Significant reduction in peak heat release rate; improvement in flame retardancy index, mitigated the loss of performance after UV aging (>60% reduction in degradation), enhanced thermal stability, and multifunctionality, including formaldehyde adsorption.	Excessive NS content leads to particle agglomeration, uneven dispersion, microcracking, and deterioration in flame-retardant and UV-resistant performance.	[147]
5	High-alumina refractory castables for intermediate-temperature industrial applications (≈800–1200 °C), particularly in petrochemical units, non-ferrous metal processing, and other thermal process equipment.	Adequate workability; high green strength after curing (before firing); low chemically bound water; high mechanical strength and elastic stability after firing (800–1250 °C); good thermal shock resistance under large temperature gradients; dimensional stability with controlled shrinkage at elevated temperatures.	Reactive silica source that promotes formation of a homogeneous amorphous aluminosilicate gel; contributes to liquid-phase formation during firing, enhancing viscous sintering and densification; influences phase evolution, favoring nepheline formation in Na systems and kalsilite/leucite in K systems; modulates viscosity and quantity of the high-temperature liquid phase, strongly affecting shrinkage and microcracking behavior.	Cement-free bonding system, avoiding drawbacks of calcium aluminate cement; high green mechanical strength; improved densification and strength after firing, especially in Na-based systems; excellent thermal shock resistance; potential for lower CO_2_ footprint and reduced drying risks.	Excess liquid-phase formation in Na-based geopolymer systems can lead to higher shrinkage, microcracking, and reduced structural reliability near 1250 °C; use of highly alkaline liquids (NaOH/KOH with NS) raises handling and safety considerations; K-based systems, while more thermally stable, show lower densification and strength than Na-based counterparts.	[148]
6	Self-compacting geopolymer concrete for structural elements, including reinforced beams.	High flowability without segregation; enhanced mechanical strength; improved durability against chloride, acid, and sulfate attack; and reliable structural performance under bending loads.	NS refines the geopolymer matrix by accelerating geopolymerization, densifying the ITZ, enhancing gel formation, and reducing pore connectivity.	Increased compressive, tensile, and flexural strength; reduced water absorption and sorptivity; improved resistance to aggressive environments; and higher load-carrying capacity and ductility of reinforced elements.	Excessive NS content leads to particle agglomeration, reduced workability, formation of weak zones and voids, and subsequent deterioration in mechanical and durability performance.	[58]
7	Geopolymer-based stabilization of expansive soils for geotechnical applications, such as road subgrades, embankments, and foundation improvement.	Improved unconfined compressive strength, enhanced water stability, and reduced sensitivity of expansive soil to moisture-induced deformation.	NS enhances the geopolymerization process by supplying additional reactive silica, refining the pore structure, and promoting the formation of C–(A)–S–H-type gels, leading to a denser and stronger soil matrix.	Significantly increases compressive strength and water resistance, while improving microstructural compactness and interparticle bonding.	Excessive NS content leads to particle agglomeration, reduced effectiveness, and potential strength loss.	[149]
8	Geopolymers for sustainable construction, particularly for solidification/stabilization of municipal solid waste incineration fly ash and fast-setting repair or prefabricated materials.	Rapid setting, high early-age and long-term compressive strength, dense microstructure, and effective immobilization of hazardous heavy metals with low leaching potential.	NS accelerates geopolymerization, shortens setting time, enhances early strength, refines pore structure, and improves heavy-metal immobilization through filling, nucleation, and pozzolanic effects.	Significant improvement in early mechanical performance, reduced porosity and permeability, enhanced formation of C–S–H/C–A–S–H gels, and increased conversion of heavy metals into stable chemical forms.	Excessive NS dosage can cause particle agglomeration, reduced dispersion efficiency, and diminishing strength gains, making dosage optimization and proper dispersion critical for practical application.	[139]
9	Underwater construction and repair applications, such as marine structures, bridge piers, ports, and foundations, where casting and curing take place directly in water.	High compressive strength under underwater curing conditions, resistance to wash-out during placement, sufficient self-compacting ability, low water permeability, retention of mechanical performance comparable to specimens cured at room conditions.	NS improved mechanical and physical performance, with the most pronounced effects observed at 0.5 wt.%. NS increased compressive strength and ultrasonic pulse velocity, and reduced capillary water absorption, indicating a denser geopolymer matrix and accelerated geopolymerization.	Significant increase in compressive strength; high strength retention under underwater curing; improved resistance to wash-out, reflected by lower pH values. Reduced capillary water absorption due to a more compact microstructure. Enhanced formation of binding phases (C-A-S-H and N-A-S-H).	Long-term durability under aggressive marine environments was not addressed. The results are specific to GGBS-based geopolymer mortar and may not be directly transferable to other geopolymer precursor systems.	[150]
10	Simulated lunar soil-based geopolymer for in situ construction of lunar infrastructure, including load-bearing elements of lunar research stations under ISRU constraints.	High early-age and long-term mechanical strength, low porosity and crack sensitivity, efficient water utilization and recovery, good flowability for molding/3D printing, and stability under extreme lunar thermal and vacuum conditions.	NS enhances geopolymerization through nucleation and chemical bonding effects, densifies the microstructure by pore filling, improves reaction homogeneity, and significantly increases compressive and flexural strength.	Strength enhancement, reduced total porosity and coarse pores, improved crack resistance and thermal stability, higher mass-strength efficiency, and reduced dependence on Earth-supplied materials while maintaining high water recovery efficiency.	Excessive NS addition leads to nanoparticle agglomeration, increased defects and coarse pores, reduced workability, and deterioration in mechanical performance.	[57]
11	Structural health monitoring of smart and low-carbon civil infrastructure elements (e.g., beams, slabs, and precast components) requiring in situ damage and strain sensing.	High mechanical strength and toughness, stable electrical conductivity, and ultra-high strain-sensing sensitivity with reliable signal response under flexural loading.	NS is critical for refining the nanopore structure, enhancing ionic conductivity through interconnected gel pores, and stabilizing the electrical sensing signal during deformation.	Synergistic improvement in sensitivity (very high gauge factor), crack detection at early stages, good mechanical performance, and reduced carbon footprint.	Sensitivity to mixture design and raw-material variability, complexity of ensuring uniform dispersion, and challenges in large-scale, field-friendly manufacturing and standardization.	[127]
12	3D-printable geopolymer mortars based on construction and demolition waste (CDW), intended for additive manufacturing of structural and non-structural building elements.	Adequate printability (extrudability, buildability, shape retention) combined with sufficient mechanical strength, controlled shrinkage-induced cracking, low permeability, and mitigation of efflorescence under ambient curing.	NS refines the pore structure and acts as a nucleation agent, promoting additional geopolymer gel formation and resulting in higher compressive and flexural strength as well as reduced permeability and efflorescence.	Densification of the geopolymer matrix; improved mechanical performance; reduced alkali migration and surface efflorescence; enhanced durability of CDW-based 3D-printed geopolymers.	NS alone does not prevent shrinkage-induced cracking in 3D-printed filaments; effective crack control requires synergistic use with other additives (e.g., methyl cellulose or calcium aluminate cement) and careful control of water demand and reaction kinetics.	[83]
13	Soft-magnetic construction materials, particularly soft-magnetic layers in airport pavements for induction heating systems (e.g., snow and ice melting), as well as other building applications requiring electromagnetic functionality combined with structural capacity.	The material must exhibit high magnetic permeability, low coercivity, and low hysteresis and eddy current losses under alternating magnetic fields, while simultaneously maintaining adequate mechanical strength and a dense, durable microstructure.	NS acts as a functional carrier and nucleation promoter by improving the dispersion of Fe_3_O_4_ nanoparticles, enhancing geopolymerization through nucleation and nano-filling effects, and facilitating a more uniform microstructure that supports both mechanical and electromagnetic performance.	The nano-SiO_2_@Fe_3_O_4_ magnetofluid enables a balanced improvement in electromagnetic and mechanical properties, leading to reduced coercivity, enhanced-saturation magnetization, improved geopolymer gel formation, and an optimized pore structure at an appropriate magnetofluid concentration.	Excessive magnetofluid content increases water demand and pore formation, which can limit further strength gains and deteriorate microstructural compactness; therefore, precise control of nano-SiO_2_@Fe_3_O_4_ dosage and dispersion is essential to avoid diminishing returns.	[49]

## Data Availability

No new data were created or analyzed in this study. Data sharing is not applicable to this article.

## Abbreviations

The following abbreviations are used in this manuscript:

ANFIS	Adaptive Neuro-Fuzzy Inference System
ANN	Artificial Neural Network
CDW	Construction and demolition waste
CNTs	Carbon nanotubes
EDS	Energy-Dispersive X-ray Spectroscopy
GGBFS	Ground granulated blast furnace slag
ISRU	In Situ Resource Utilization
ITZ	Interfacial Transition Zone
MIP	Mercury Intrusion Porosimetry
NMR	Nuclear Magnetic Resonance
NS	Nano-silica
OPC	Ordinary Portland Cement
PVA	Polyvinyl alcohol
SCGC	Self-compacting geopolymer concrete
SEM	Scanning Electron Microscopy
SF	Silica fume
XRD	X-ray Diffraction
UPV	Ultrasonic pulse velocity
